# Efficient full-colour organic light-emitting diodes based on donor–acceptor electroluminescent materials with a reduced singlet–triplet splitting energy gap†

**DOI:** 10.1039/c8ra09486a

**Published:** 2019-01-23

**Authors:** Jayaraman Jayabharathi, Ramaiyan Ramya, Venugopal Thanikachalam, Palanivel Jeeva, Elayaperumal Sarojpurani

**Affiliations:** Department of Chemistry, Annamalai University Annamalai Nagar Tamilnadu 608 002 India; Sri Manakula Vinayagar Engineering College India

## Abstract

A series of efficient blue-emitting materials, namely, Cz-DPVI, Cz-DMPVI, Cz-DEPVI and TPA-DEPVI, possessing a donor–acceptor architecture with dual carrier transport properties and small singlet–triplet splitting is reported. These compounds exhibit excellent thermal properties with a very high glass-transition temperature (*T*_g_), and thus, a stable uniform thin film was formed during device fabrication. Among the weak donor compounds, specifically, Cz-DPVI, Cz-DMPVI and Cz-DEPVI, the Cz-DEPVI-based device showed the maximum efficiencies (*L*: 13 955 cd m^−2^, *η*_ex_: 4.90%, *η*_c_: 6.0 cd A^−1^, and *η*_p_: 5.4 lm W^−1^) with CIE coordinates of (0.15, 0.06) at 2.8 V. The electroluminescent efficiencies of Cz-DEPVI were higher than that of the strong donor TPA-DEPVI-based device (*L*: 13 856 cd m^−2^, *η*_ex_: 4.70%, *η*_c_: 5.7 cd A^−1^, and *η*_p_: 5.2 lm W^−1^). Furthermore, these blue emissive materials were used as hosts to construct efficient green and red phosphorescent OLEDs. The green device based on Cz-DEPVI:Ir(ppy)_3_ exhibited the maximum *L* of 8891 cd m^−2^, *η*_ex_ of 19.3%, *η*_c_ of 27.9 cd A^−1^ and *η*_p_ of 33.4 lm W^−1^ with CIE coordinates of (0.31, 0.60) and the red device based on Cz-DEPVI:Ir(MQ)_2_(acac) exhibited the maximum *L* of 40 565 cd m^−2^, *η*_ex_ of 19.9%, *η*_c_ of 26.0 cd A^−1^ and *η*_p_ of 27.0 lm W^−1^ with CIE coordinates of (0.64, 0.37).

## Abstract 2

The Cz-DEPVI device showed high efficiencies of *L*: 13955 cd m^−2^, *η*_ex_: 4.90%, *η*_c_: 6.0 cd A^−1^, *η*_p_: 5.4 lm W^−1^ and CIE coordinates of (0.15, 0.06) at 2.8 V.

## Graphical abstract

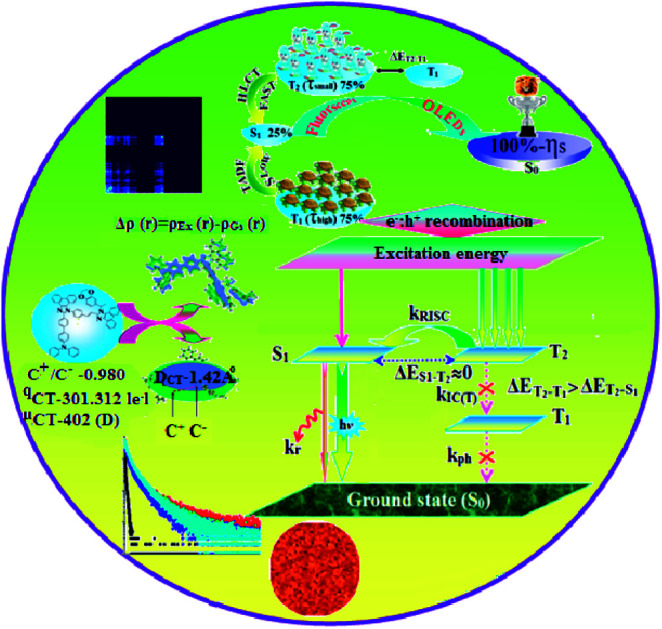

## 1. Introduction

**Scheme 1:**
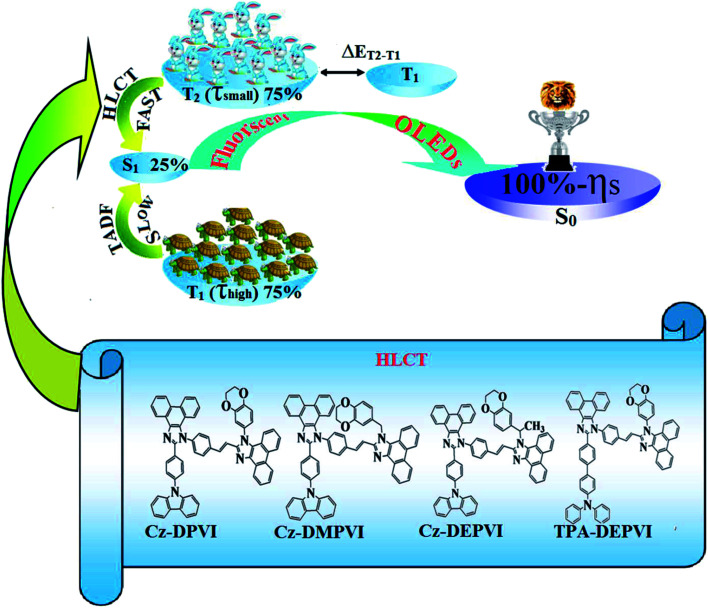
Effect of TADF and HLCT on 100% exciton utilization efficiency (*η*_S_).

Currently, blue organic light-emitting devices (OLEDs) have attracted increasing interest due to their applications in FPD (flat panel displays) and SSL (solid state lighting).^1–3^ However, the development of high-performance blue emissive materials with high fluorescent quantum yield (*Φ*), high thermal stability (*T*_d_ and *T*_g_), good film-forming ability and balanced carrier injection/transporting properties remains challenging.^4–6^ Two important factors have been reported for 100% exciton utilization efficiency (*η*_S_): (i) thermally activated delayed fluorescence (TADF) and (ii) hybrid local and charge transfer (HLCT) state (Scheme 1).^7^ Although high external quantum efficiencies have been obtained from blue (37.5%), green (31.3%) and red (17.5%) TADF OLEDs, their fabrication is expensive since the long lifetime of the T1 state in the TADF process suffers from exciton annihilation.^8,9^ Organic donor–acceptor (D–A) compounds with hybridized local and charge transfer exhibit high *η*_S_ in fluorescent OLEDs, which can be attributed to the hot exciton mechanism.^10–16^ When the LE and CT states are close in energy, mixing of the LE and CT states is possible as a linear combination of both states (*ψ*(CT) and *ψ*(LE), *i.e.*, *Ψ*(S_1_) = *Ψ*(LE) + *λ* × *Ψ*(CT)) and *λ* = |〈*Ψ*_LE_|*H*|*Ψ*_CT_〉/*E*_CT_ − *E*_LE_|. The low-lying LE-dominated HLCT state (high % LE) provides a high radiative rate (*k*_r_) for high photoluminescence efficiency (*η*_PL_); whereas, the high-lying CT (high % CT) dominated HLCT state is responsible for high *η*_S_ through the RISC process together with the hot exciton mechanism.^14–17^

The external quantum efficiency (*η*_EQE_) and exciton utilization efficiency (*η*_S_) can be calculated as follows: *η*_EQE_ = *η*_out_ × *η*_IQE_ =*η*_out_ × *η*_rec_ × *η*_γ_ × *Φ*_PL_ and *η*_S_ = *η*_out_ × *η*_rec_ × *η*_PL_, where, *η*_IQE_ is the internal quantum efficiency, *η*_out_ the light outcoupling efficiency (1/2*n*^2^, *n* = 1.5, *η*_out_ ∼ 20%), *η*_rec_ the efficiency for electron hole recombination (100%), *η*_PL_ the photoluminescence efficiency of the film and *η*_S_ the exciton utilization efficiency.^18^ Presently, the construction of emissive states consisting of both LE and CT components for high PL efficiency (*η*_PL_: LE state) and high exciton utilization (*η*_S_: from CT state) is interest.^19^ According to the energy gap law, a larger energy gap between the T_2_ and T_1_ states reduces the internal conversion (IC) 
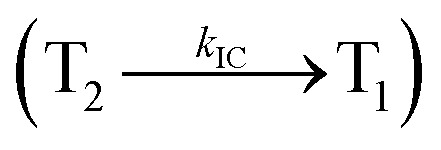
, which results in hot RISC 
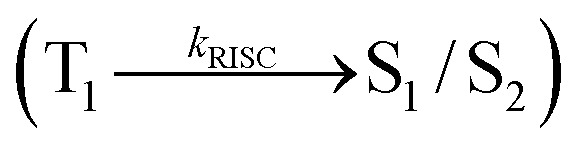
 rather than cold RISC 
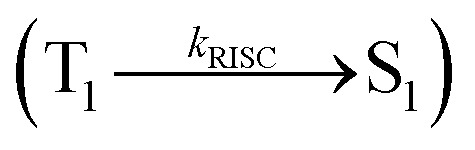
.^20^ Therefore, the hot exciton mechanism with the HLCT state increases the *η*_EQE_ as a result of the coexistence of high *η*_PL_ and high *η*_S_. A blue emissive material with balanced carrier transport characteristics and high triplet energy (*E*_T_) may be employed as a host for green and red phosphorescent emitters. However, high-performance non-doped blue electroluminescent materials are not a suitable host for phosphorescent OLEDs due to their low *E*_T_ and poor carrier transport properties.^3^ An efficient host for green and red phosphors exhibits low efficiency when used as blue OLEDs.^21^ Therefore, it is still challenging to achieve efficient full-color OLEDs with blue emissive materials. Herein, we report the use of multi-functional organic OLED materials (Cz-DPVI, Cz-DMPVI, Cz-DEPVI and TPA-DEPVI) as (i) emitters in blue OLEDs and (ii) hosts for green and red OLEDs. These materials consist of a hole transport moiety (donor) and an electron transport moiety (acceptor) with high quantum yield in film (*ϕ*_film_) with small singlet–triplet splitting (Δ_ST_) to ensure that the triplet excited state energy (*E*_T_) is high enough to excite the green and red phosphorescent dopant. The H–H repulsion of the styryl fragment (i) with a phenanthrene part and dihydrobenzodioxin and with a 9-(*p*-tolyl)-9*H*-carbazole moiety (Cz-DPVI, Cz-DMPVI and Cz-DEPVI)/phenyl of the TPA moiety (TPA-DEPVI) leads to a twisted configuration, which enhanced the twist angle, and thus, shortened the conjugation length. The solvatochromic effect of Cz-DPVI, Cz-DMPVI, Cz-DEPVI and TPA-DEPVI was studied to understand the excited state characteristics and interstate coupling strength of the LE [(D–A*)/(D*–A)] and CT [(D^+^–A^−^)] components. The LE and CT composition in the single emissive state was discussed using natural transition orbital (NTO), centroids of charges and transition density matrix (TDM) analysis. The hybridization of the LE and CT energy states was used for the molecular design and their composition in HLCT was tuned, which resulted in high EL efficiency. The weak electron-donating carbazole (Cz) in Cz-DPVI, Cz-DMPVI and Cz-DEPVI decreased the % CT with an increase in the % LE composition in the S_1_ state; whereas, in TPA-DEPVI, the extended π-conjugation (increased % LE) resulted in an enhancement in PL efficiency (*η*_PL_) while simultaneously maintaining donor strength (% LE). Among the weak donor compounds, specifically, Cz-DPVI, Cz-DMPVI and Cz-DEPVI, the Cz-DEPVI-based device showed the maximum efficiencies (*L*: 13 955 cd m^−2^, *η*_ex_: 4.90%, *η*_c_: 6.0 cd A^−1^, and *η*_p_: 5.4 lm W^−1^) with CIE coordinates of (0.15, 0.06) at 2.0 V. The electroluminescence efficiencies of Cz-DEPVI were higher than that of the strong donor TPA-DEPVI-based device (*L*: 13 856 cd m^−2^, *η*_ex_: 4.70%, *η*_c_: 5.7 cd A^−1^, and *η*_p_: 5.2 lm W^−1^). The green device based on Cz-DEPVI:Ir(ppy)_3_ exhibited the maximum *L* of 8891 cd m^−2^, *η*_ex_ of 19.3%, *η*_c_ of 27.9 cd A^−1^ and *η*_p_ of 33.4 lm W^−1^ with CIE coordinates of (0.31, 0.60) and the red device based on Cz-DEPVI:Ir(MQ)_2_(acac) exhibited the maximum *L* of 40 565 cd m^−2^, *η*_ex_ of 19.9%, of *η*_c_ of 26.0 cd A^−1^ and *η*_p_ of 27.0 lm W^−1^ with CIE coordinates of (0.64, 0.37). These results are highly useful for the design of low-cost fluorescent materials using subtle molecular modification and the HLCT emissive state principle.

## 2. Experimental

### 2.1. Measurements and general methods

**Scheme 2:**
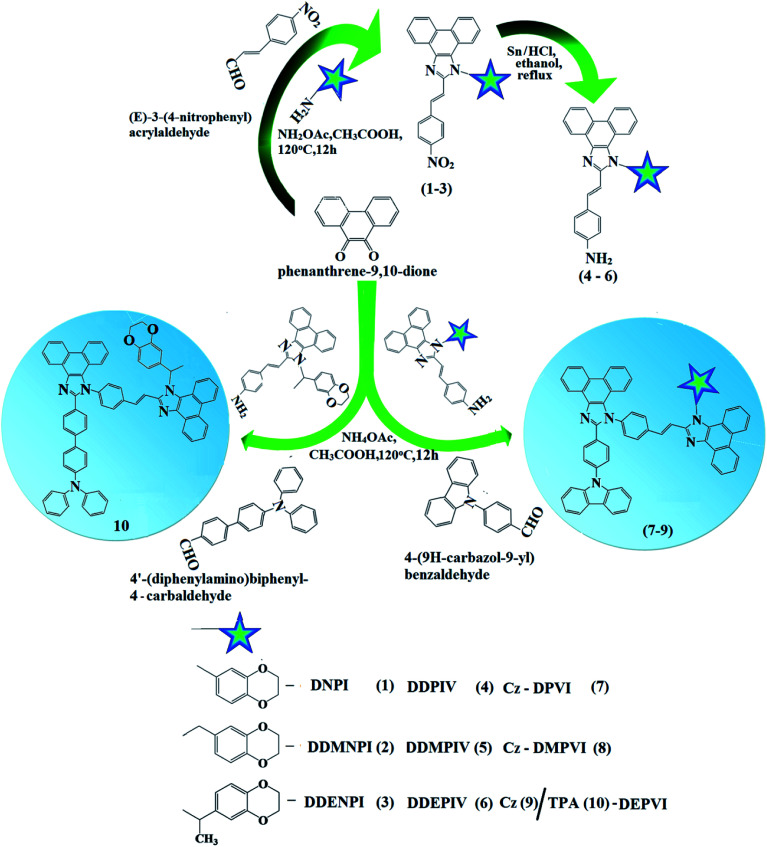
Synthetic route for Cz-DPVI, Cz-DMPVI, Cz-DEPVI and TPA-DEPVI.

The reagents and solvents used for synthesis (Scheme 2) were purchased from Sigma-Aldrich. ^1^H and ^13^C NMR spectra were recorded with a Bruker 400 MHz spectrometer and mass spectra with an Agilent spectrometer (LCMS VL SD). UV-vis absorption spectra in solution and film were measured on a Perkin-Elmer Lambda 35 and Lambda 35 spectrophotometer with an integrated sphere (RSA-PE-20), respectively. Photoluminescence (PL) studies were carried out using a PerkinElmer LS55 fluorescence spectrometer. Thermogravimetric analysis (TGA) and differential scanning calorimetry (DSC) were performed on a PerkinElmer thermal analysis system and NETZSCH-DSC-204, respectively, with a heating rate of 10 °C min^−1^ and N_2_ flow rate of 100 mL min^−1^ for both. The fluorescence lifetimes of the emissive materials were estimated from the time-resolved fluorescence decay spectra obtained *via* the time-correlated single-photon counting (TCSPC) method on a Horiba Fluorocube-01-NL lifetime system equipped with a Nano LED excitation source and TBX-PS detector. The DAS6 software was used to analyse decay *via* the reconvolution method and goodness of fit was determined using reduced *χ*^2^ values. The absolute quantum yield (PLQY) was determined using an F7100 fluorescence spectrometer. Cyclic voltammetry was performed using a potentiostat CHI 630A electrochemical analyzer (platinum electrode and platinum wire as the working electrode and counter electrode, respectively, Ag/Ag^+^ as the reference electrode, and scan rate of 100 mV s^−1^). Ferrocene was used as the internal standard with the highest occupied molecular orbital energy (HOMO) of −4.80 eV and 0.1 M tetrabutylammoniumperchlorate in CH_2_Cl_2_ as the supporting electrolyte. The HOMO energies were calculated by measuring the oxidation potentials [*E*_HOMO_ = −(*E*_ox_ + 4.8 eV)] and the LUMO energies were estimated by subtracting the optical band gap from the HOMO energies [*E*_LUMO_ = *E*_HOMO_ − 1239/*λ*_onset_].

### 2.2. Computational details

For the theoretical calculations, the ground state (DFT)/excited state (TD-DFT) geometrical properties were optimized by employing the Gaussian 09 program.^22^ The multifunctional wavefunction analyzer (Multiwfn)^22^ was used to determine the nature of the electronic transitions of the excited states and natural transition orbitals (NTOs).

### 2.3. Synthesis of (*E*)-1-(2,3-dihydrobenzo[*b*][1,4]dioxin-6-yl)-2-(4-nitrostyryl)-1*H*-phenanthro[9,10-*d*]imidazole (DDNPI)

A mixture of phenanthrenequinone (2.08 g, 10 mmol), 4-nitrocinnamaldehyde (1.51 g, 10 mmol), 1,4-benzodioxane-6-amine (4.65 g, 50 mmol) and ammonium acetate (3.08 g, 40 mmol) in acetic acid (25 mL) was refluxed (120 °C, 12 h, and N_2_ stream). The reaction mixture was cooled and poured into methanol. The separated crude product was purified by column chromatography using hexane:ethylacetate as the eluent (Scheme 2). Yield: 60%. Anal. calcd for C_31_H_21_N_3_O_4_: C, 74.53; H, 4.25; N, 8.41. Found: C, 74.50; H, 4.21; N, 8.35. ^1^H NMR (400 MHz, CDCl_3_): *δ* 4.18–4.36 (m, 4H), 6.80 (d, *J* = 16.2 Hz, 1H), 6.88 (t, 1H), 7.12 (d, *J* = 15.0 Hz, 1H), 7.28 (d, *J* = 8.0 Hz*,* 1H), 7.25–7.39 (m, 5H), 7.60–7.69 (m, 5H), 8.05 (s, 1H), 8.59 (t, 1H), 8.75 (s, 1H). ^13^C NMR (100 MHz, CDCl_3_): *δ* 64.30, 64.32, 102.53, 110.82, 114.76, 121.12, 122.42, 126.53, 127.31, 127.69, 127.94, 128.39, 131.43, 131.54, 133.39, 141.25, 141.57, 146.09, 147.61, 147.61. MS: *m*/*z*. 499.08 [M^+^]; calcd 499.26.

### 2.4. Synthesis of 4-((*E*)-2-(1-(2,3-dihydrobenzo[*b*][1,4]dioxin-6-yl)-1*H*-phenanthro[9,10-*d*]imidazol-2-yl)vinyl)benzenamine (DDPIV)

A mixture of (*E*)-1-(2,3-dihydrobenzo[*b*][1,4]dioxin-6-yl)-2-(4-nitrostyryl)-1*H*-phenanthro[9,10-*d*]imidazole (DDNPI) (4.15 g, 10 mmol) and 10% Sn/HCl (250 mg) in 25 mL ethanol was refluxed under stirring and 80% hydrazine hydrate (15 mL) was added dropwise within 30 min and stirring was continued for 14 h. The reaction mixture was neutralized with aq. HCl and the obtained white product was recrystallized from an ethanol : water mixture. Yield: 58%. Anal. calcd for C_31_H_23_N_3_O_2_: C, 79.31; H, 4.92; N, 8.94. Found: C, 79.28; H, 4.88; N, 8.89. ^1^H NMR (400 MHz, CDCl_3_) *δ* 4.01 (s, 2H), 4.10–4.30 (m, 4H), 6.71 (d, *J* = 16.0 Hz, 1H), 6.52 (s, 2H), 6.95 (d, *J* = 8.2 Hz, 1H), 6.98 (d, *J* = 16.2 Hz, 1H), 6.85–6.93 (m, 6H), 7.05 (s, 2H), 7.82 (d, *J* = 8.2 Hz, 1H), 8.13 (d, *J* = 8.4 Hz, 2H), 8.90 (d, *J* = 7.6 Hz*,* 2H). ^13^C NMR (100 MHz, CDCl_3_): *δ* 63.35, 101.54, 112.82, 115.93, 116.20, 122.43, 125.21, 126.01, 126.22, 126.56, 126.82, 127.63, 128.35, 131.41, 133.43, 141.49, 146.21, 147.60, 147.21. MS: *m*/*z*. 469.10 [M^+^]; calcd 469.26.

### 2.5. Synthesis of 2-(4-(9*H*-carbazol-9-yl)phenyl)-1-(4-((*E*)-2-(1-(2,3-dihydrobenzo[*b*][1,4] dioxin-8-yl)-1*H*-phenanthro[9,10-*d*]imidazol-2-yl)vinyl)phenyl)-1*H*-phenanthro[9,10-*d*] imidazole (Cz-DPVI)

A mixture of phenanthrenequinone (0.416 g, 2 mmol), 4-(9*H*-carbazol-9-yl)benzaldehyde (0.698 g, 2 mmol), 4-((*E*)-2-(1-(2,3-dihydrobenzo[*b*]rdioxin-6-yl)-1*H*-phenanthro[9,10-*d*]imidazol-2-yl)vinyl)benzenamine (DDPIV) (1.16 g, 3 mmol) and ammonium acetate (1.54 g, 20 mmol) in glacial acetic acid (25 mL) was refluxed (140 °C, 20 h, and N_2_ stream).^23–25^ The reaction mixture was poured into methanol and the separated white solid was filtered, washed with water and purified by column chromatography using CH_2_Cl_2_ as the eluent. Yield: 60%. Anal. calcd for C_64_H_41_N_5_O_2_: C, 84.28; H, 4.52; N, 7.65. Found: C, 84.19; H, 4.48; N, 7.31. ^1^H NMR (400 MHz, CDCl_3_) *δ* 4.18–4.32 (m, 4H), 6.73 (d, *J* = 15.2 Hz, 2H), 6.70 (s, 1H), 6.99 (s, 2H), 7.01–7.50 (m, 8H), 7.05–7.41 (m, 8H), 7.62–7.72 (m, 10H), 7.54 (d, *J* = 16.2 Hz, 2H), 8.33 (d, *J* = 7.8 Hz, 2H), 8.65 (t, 2H). ^13^C NMR (100 MHz, CDCl_3_): *δ* 64.21, 101.32, 111.10, 119.21, 120.01, 122.21, 122.35, 122.42, 126.01, 126.21, 126.43, 126.60, 127.31, 128.13, 128.30, 129.60, 131.54, 133.41, 139.71, 141.31, 146.21, 147.65. MS: *m*/*z*. 910.29 [M^+^]; calcd 910.35.

### 2.6. Synthesis of (*E*)-1-((2,3-dihydrobenzo[*b*][1,4]dioxin-6-yl)methyl)-2-(4-nitrostyryl)-1*H*-phenanthro[9,10-*d*]imidazole (DDMNPI)

DDMNPI was synthesized using (2,3-dihydrobenzo[*b*][1,4]dioxin-6-yl)methanamine following a procedure similar to that of DDNPI. Yield: 62%. Anal. calcd for C_32_H_23_N_3_O_4_: C, 74.82; H, 4.50; N, 8.17. Found: C, 74.78; H, 4.41; N, 8.10. ^1^H NMR (400 MHz, CDCl_3_): *δ* 4.36 (m, 4H), 4.99 (s, 2H) 6.46 (d, *J* = 16.4 Hz, 1H), 6.51 (d, *J* = 8.0 Hz, 1H), 7.13 (d, *J* = 15.0 Hz, 1H), 7.25 (d, *J* = 8.0 Hz*,* 1H), 7.24–7.40 (m, 5H), 7.62–7.65 (m, 5H), 8.15 (s, 1H), 8.68 (t, 1H), 8.79 (s, 1H). ^13^C NMR (100 MHz, CDCl_3_): *δ* 50.2, 64.32, 102.58, 110.85, 114.67, 121.19, 122.48, 126.57, 126.32, 127.30, 127.65, 131.49, 131.54, 133.37, 141.29, 141.67, 146.18, 147.63, 147.68. MS: *m*/*z*. 513.10 [M^+^]; calcd 513.19.

### 2.7. Synthesis of (*E*)-4-(2-(1-((2,3-dihydrobenzo[*b*][1,4]dioxin-6-yl)methyl)-1*H*-phenanthro[9,10-*d*]imidazol-2-yl)vinyl)aniline (DDMPIV)

DDMPIV was synthesized using DDMNPI following a procedure similar to that of DDMNPI. Yield: 60%. Anal. calcd for C_32_H_25_N_3_O_2_: C, 79.48; H, 5.22; N, 8.69. Found: C, 79.32; H, 5.18; N, 8.58. ^1^H NMR (400 MHz, CDCl_3_) *δ* 4.05 (s, 2H), 4.39 (m, 4H), 4.99 (s, 2H), 6.46 (d, *J* = 16.0 Hz, 1H), 6.53 (s, 2H), 6.89 (d, *J* = 8.0 Hz, 1H), 6.99 (d, *J* = 16.2 Hz, 1H), 6.75–6.98 (m, 6H), 7.05 (s, 2H), 7.84 (d, *J* = 16.0 Hz, 1H), 8.14 (d, *J* = 8.0 Hz, 2H), 8.95 (d, *J* = 7.0 Hz, 2H). ^13^C NMR (100 MHz, CDCl_3_): *δ* 50.25, 63.38, 112.85, 114.93, 116.25, 121.38, 126.31, 126.43, 126.52, 126.56, 126.72, 127.73, 127.85, 128.41, 131.43, 133.49, 141.23, 143.60, 146.21, 147.28 MS: *m*/*z*. 483.53 [M^+^]; calcd 483.62.

### 2.8. Synthesis of 2-(4-(9*H*-carbazol-9-yl)phenyl)-1-(4-((*E*)-2-(1-((2,3-dihydrobenzo[*b*][1,4]dioxin-6-yl)methyl)-1*H*-phenanthro[9,10-*d*]imidazol-2-yl)vinyl)phenyl)-1*H*-phenanthro[9,10-*d*]imidazole (Cz-DMPVI)

Cz-DMPVI was synthesized using DDMNPI following a procedure similar to that of Cz-DPVI. Yield: 58%. Anal. calcd for C_65_H_43_N_5_O_2_: C, 84.28; H, 4.52; N, 7.65. Found: C, 84.19; H, 4.48; N, 7.31. ^1^H NMR (400 MHz, CDCl_3_) *δ* 4.32 (m, 4H), 6.46 (s, 1H), 6.99 (d, 1H), 6.75 (d, *J* = 16.0 Hz, 2H), 6.68 (s, 1H), 6.95 (s, 2H), 7.02–7.59 (m, 9H), 7.05–7.48 (m, 7H), 7.60–7.79 (m, 10H), 7.56 (d, *J* = 8.8 Hz, 2H), 8.38 (d, *J* = 8.0 Hz, 2H), 8.67 (t, 2H). ^13^C NMR (100 MHz, CDCl_3_): *δ* 50.29, 63.98, 111.12, 111.19, 120.01, 122.25, 122.38, 122.46, 126.01, 126.22, 126.44, 126.62, 127.32, 128.15, 128.38, 129.65, 131.59, 133.39, 139.89, 141.38, 146.27, 147.68. MS: *m*/*z*. 925.27 [M^+^]; calcd 925.38.

### 2.9. Synthesis of (*E*)-1-(1-(2,3-dihydrobenzo[*b*][1,4]dioxin-6-yl)ethyl)-2-(4-nitrostyryl)-1*H*-phenanthro[9,10-*d*]imidazole (DDENPI)

DDENPI was synthesized using 1-(2,3-dihydrobenzo[*b*][1,4]dioxin-6-yl)ethanamine following a procedure similar to that of DDNPI. Yield: 62%. Anal. calcd for C_33_H_25_N_3_O_4_: C, 75.12; H, 4.75; N, 7.93. Found: C, 75.01; H, 4.68; N, 7.87. ^1^H NMR (400 MHz, CDCl_3_): *δ* 1.90 (s, 3H), 4.38 (m, 4H), 5.16 (d, *J* = 8.8 Hz*,* 1H), 6.53 (d, *J* = 15.0 Hz, 1H), 6.51 (d, *J* = 8.0 Hz, 1H), 7.14 (d, *J* = 7.0 Hz, 1H), 7.28 (d, *J* = 8.0 Hz, 1H), 7.34–7.56 (m, 6H), 7.68–7.68 (m, 4H), 8.12 (s, 1H), 8.14 (t, 1H), 8.89 (s, 1H). ^13^C NMR (100 MHz, CDCl_3_): *δ* 22.31, 54.01, 64.33, 112.53, 112.82, 114.98, 121.02, 121.09, 122.43, 126.61, 126.68, 126.73, 131.53, 133.49, 136.68, 141.33, 141.58, 143.93, 146.63, 147.65. MS: *m*/*z*. 527.12 [M^+^]; calcd 527.01.

### 2.10. Synthesis of (*E*)-4-(2-(1-(1-(2,3-dihydrobenzo[*b*][1,4]dioxin-6-yl)ethyl)-1*H*-phenanthro[9,10-*d*]imidazol-2-yl)vinyl)aniline (DDEPIV)

DDEPIV was synthesized using DDENPI following a procedure similar to that of DDMNPI. Yield: 50%. Anal. calcd for C_33_H_27_N_3_O_2_: C, 79.63; H, 5.15; N, 6.57. Found: C, 79.58; H, 5.08; N, 6.48. ^1^H NMR (400 MHz, CDCl_3_): *δ* 1.83 (s, 3H), 4.03 (s, 2H), 4.37 (m, 4H), 5.17 (d, *J* = 8.0 Hz, 1H), 6.50 (d, *J* = 16.0 Hz, 1H), 6.54 (d, *J* = 8.6 Hz, 1H), 7.18 (d, *J* = 8.0 Hz, 1H), 7.29 (d, *J* = 16.0 Hz*,* 1H), 7.34–7.56 (m, 4H), 7.65–7.77 (m, 6H), 8.10 (s, 1H), 8.18 (t, 1H), 8.80 (s, 1H). ^13^C NMR (100 MHz, CDCl_3_): *δ* 21.99, 53.01, 63.98, 112.58, 112.86, 114.97, 121.05, 121.10, 122.45, 126.60, 126.65, 126.78, 131.54, 133.47, 136.65, 141.34, 141.56, 143.90, 146.67, 147.69. MS: *m*/*z*. 497.18 [M^+^]; calcd 497.27.

### 2.11. Synthesis of 2-(4-(9*H*-carbazol-9-yl)phenyl)-1-(4-((*E*)-2-(1-(1-(2,3-dihydrobenzo[*b*][1,4]dioxin-7-yl)ethyl)-1*H*-phenanthro[9,10-*d*]imidazol-2-yl)vinyl)phenyl)-1*H*-phenanthro[9,10-*d*] imidazole (Cz-DEPVI)

Cz-DEPVI was synthesized using DDMNPI following a procedure similar to that of Cz-DPVI yield: 50%. Anal. calcd for C_66_H_45_N_5_O_2_: C, 84.32; H, 4.80; N, 7.42. Found: C, 84.25; H, 4.75; N, 7.35. ^1^H NMR (400 MHz, CDCl_3_) *δ* 1.88 (s, 3H), 4.33 (m, 4H), 5.15 (d, *J* = 8.0 Hz, 1H), 6.57 (s, 1H), 6.95 (d, 1H), 6.78 (d, *J* = 15.0 Hz, 2H), 6.65 (s, 1H), 6.93 (s, 2H), 7.01–7.55 (m, 10H), 7.05–7.45 (m, 7H), 7.63–7.81 (m, 14H), 7.58 (d, *J* = 8.0 Hz, 2H), 8.39 (d, *J* = 8.8 Hz, 2H), 8.63 (t, 2H). ^13^C NMR (100 MHz, CDCl_3_): *δ* 21.98, 53.29, 64.01, 111.13, 111.18, 120.02, 122.29, 122.35, 122.45, 126.09, 126.28, 126.44, 126.66, 127.33, 128.11, 128.37, 129.66, 131.55, 133.35, 139.88, 141.37, 146.22, 147.63. MS: *m*/*z*. 938.38 [M^+^]; calcd 938.45.

### 2.12. (*E*)-4′-(1-(4-(2-(1-((2,3-Dihydrobenzo[*b*][1,4]dioxin-6-yl)ethyl)-1*H*-phenanthro[9,10-imidazol-2-yl)vinyl)phenyl)-1*H*-phenanthro[9,10-*d*]imidazol-2-yl)-*N*,*N*-diphenyl-[1,1′-biphenyl]-4-amine (TPA-DEPVI)

A mixture of phenanthrenequinone (0.416 g, 2 mmol), 4′-(diphenylamino)biphenyl-4-carbaldehyde (0.698 g, 2 mmol), (DDMNPI) (1.20 g, 3 mmol), ammonium acetate (1.54 g, 20 mmol) and glacial acetic acid (25 mL) was refluxed at 120 °C for 12 h under a nitrogen atmosphere.^23–25^ The reaction mixture was poured into methanol and the separated white solid was filtered, washed with water and purified by column chromatography using CH_2_Cl_2_ as the eluent. Yield: 52% Anal. calcd for C_72_H_51_N_5_O_2_: C, 84.90; H, 5.01; N, 6.84. Found: C, 84.78; H, 4.98; N, 6.79. ^1^H NMR (400 MHz, CDCl_3_) *δ* 1.86 (s, 3H), 4.30 (m, 4H), 4.10 (s, 3H), 6.62 (d, *J* = 16.0 Hz, 2H), 6.73 (s, 3H), 6.86 (d, *J* = 15.0 Hz, 1H), 7.06–7.23 (m, 21H), 7.48 (d, *J* = 7.6 Hz, 2H), 7.62–7.66 (m, 10H), 8.24 (d, *J* = 8.2 Hz, 1H), 8.61 (t, 1H). ^13^C NMR (100 MHz, CDCl_3_): *δ* 20.98, 63.21, 114.17, 123.79, 123.88, 124.75, 125.74, 126.27, 126.49, 127.37, 128.09, 128.81, 129.08, 129.54, 129.83, 130.04, 130.29, 131.89, 132.06, 134.94, 143.65, 150.17. MALDI-TOF MS: *m*/*z*. 1017.28 [M^+^]; calcd 1017.34.

### 2.13. Device fabrication and measurement

Devices with the configuration of (i) [ITO/NPB (60 nm)/Cz-DPVI/Cz-DMPVI/Cz-DEPVI/TPA-DEPVI (20 nm)/Alq_3_ (30 nm)/LiF (1 nm)/Al (100 nm)], (ii) hole-only device: [ITO/HATCN (10 nm)/NPB (20 nm)/Cz-DPVI/Cz-DMPVI/Cz-DEPVI/TPA-DEPVI (60 nm)/NPB (20 nm)/Al(100 nm)], (iii) electron-only device: [ITO/TPBi (10 nm)/Cz-DPVI/Cz-DMPVI/Cz-DEPVI/TPA-DEPVI (60 nm)/TPBi (10 nm)/LiF (1 nm)/Al (100 nm)], (iv) green device: ITO/NPB (40 nm)/TCTA (5 nm)/Cz-DPVI (30 nm): 5 wt% Ir(ppy)_3_/Cz-DMPVI (30 nm): 5 wt% Ir(ppy)_3_/Cz-DEPVI (30 nm): 5 wt% Ir(ppy)_3_/TPA-DEPVI (30 nm): 5 wt% Ir(ppy)_3_/TPBI (50 nm)/LiF (1 nm)/Al (100 nm)] and (v) red device: ITO/NPB (40 nm)/TCTA (5 nm)/Cz-DPVI (30 nm): 8 wt% Ir(MQ)_2_(acac)/Cz-DMPVI (30 nm): 8wt% Ir(MQ)_2_(acac)/Cz-DEPVI (30 nm): 8 wt% Ir(MQ)_2_(acac)/TPA-DEPVI (30 nm): 8wt% Ir(MQ)_2_(acac)/TPBI (50 nm)/LiF (1 nm)/Al (100 nm)] were fabricated on pre-cleaned ITO-coated glass substrates with a resistance of 20 Ω sq^−1^. Current density–voltage characteristics were measured with a Keithley 2400 power source. EL spectra and CIE coordinates were recorded with a spectrometer (USB-650-VIS-NIR, Ocean Optics, Inc, USA).

## 3. Results and discussion

Efficient blue emitters, namely, 2-(4-(9*H*-carbazol-9-yl)phenyl)-1-(4-((*E*)-2-(1-(2,3-dihydrobenzo[*b*][1,4]dioxin-8-yl)-1*H*-phenanthro[9,10-*d*]imidazol-2-yl)vinyl)phenyl)-1*H*-phenanthro[9,10-*d*] imidazole (Cz-DPVI), 2-(4-(9*H*-carbazol-9-yl)phenyl)-1-(4-((*E*)-2-(1-((2,3-dihydrobenzo[*b*][1,4]dioxin-6-yl)methyl)-1*H*-phenanthro[9,10-*d*]imidazol-2-yl)vinyl) phenyl)-1*H*-phenanthro [9,10-*d*]imidazole (Cz-DMPVI), 2-(4-(9*H*-carbazol-9-yl)phenyl)-1-(4-((*E*)-2-(1-(1-(2,3-dihydrobenzo[*b*][1,4]dioxin-7-yl)ethyl)-1*H*-phenanthro[9,10-*d*]imidazol-2-yl)vinyl)phenyl)-1*H*-phenanthro[9,10-*d*]imidazole (Cz-DEPVI) and 4′-(1-(4-(2-(1-(2,3-dihydrobenzo[*b*][1,4]dioxin-6-yl)-1*H*-phenanthro[9,10-*d*]imidazol-2-yl)vinyl)phenyl)-1*H*-phenanthro[9,10-*d*]imidazol-2-yl)-*N*,*N*-diphenyl-[1,1′-biphenyl]-4-amine (TPA-DEPVI) were synthesized in appreciable yields of 60%, 58%, 50% and 52% and characterized *via*^1^H and ^13^C NMR, high resolution mass spectroscopy and elemental analysis. The synthetic route for the emissive materials is displayed in Scheme 2. The weak electron-donating carbazole in Cz-DPVI, Cz-DMPVI and Cz-DEPVI increased the % LE component in the emissive state (S_1_ HLCT), which resulted in a high photoluminance efficiency (*η*_PL_).

### 3.1. Potential energy surface (PES) scan studies and thermal properties

**Fig. 1:**
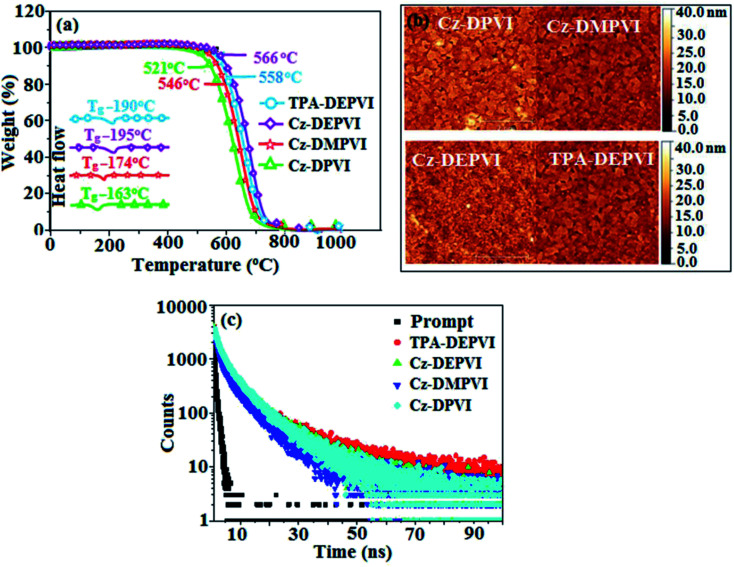
(a) TGA (inset: DSC) graph, (b) AFM images and (c) lifetime spectra of Cz-DPVI, Cz-DMPVI, Cz-DEPVI and TPA-DEPVI.

The effect of the configuration of the emissive materials on the photophysical properties of Cz-DPVI, Cz-DMPVI, Cz-DEPVI and TPA-DEPVI was investigated theoretically using DFT/B3LYP/6-31G (d) level (Fig. S1†). A potential energy surface scan for C67–C109–C58–C57 (Cz-DPVI)/C67–C109–C58–C57 (Cz-DMPVI)/C67–C109–C58–C57 (Cz-DEPVI)/C67–C109–C58–C57 (TPA-DEPVI) was performed, where the geometrical parameters were relaxed, and the torsional angles were altered in steps of 0°, 20°, 40°, 60°…360°. The minimum energy conformation in the PES of Cz-DPVI, Cz-DMPVI, Cz-DEPVI and TPA-DEPVI showed that the side chain (*E*)-1-(2,3-dihydrobenzo[*b*][1,4]dioxin-6-yl)-2-(4-styryl)-1*H*-phenanthro[9,10-*d*]imidazole (Cz-DPVI)/(*E*)-1-((2,3-dihydrobenzo[*b*][1,4]dioxin-6-yl)methyl)-2-styryl-1*H*-phenanthro[9,10-*d*]imidazole (Cz-DMPVI)/(*E*)-1-(1-(2,3-dihydrobenzo[*b*][1,4]dioxin-6-yl)ethyl)-2-styryl-1*H*-phenanthro[9,10-*d*] imidazole (Cz-DEPVI and TPA-DEPVI) was orthogonally attached to the imidazole nitrogen atom (N23) by ∼90.0°. The orthogonal dihedral angles (∼90.0°) between the side chain at N(23) and the substituent at main frame C(25), *i.e.*, the 2-(4-(9*H*-carbazol-9-yl)phenyl)-1-methyl-1*H*-phenanthro[9,10-*d*]imidazole core (Cz-DPVI, Cz-DMPVI, Cz-DEPVI and TPA-DEPVI)/*N*,*N*-diphenyl-[1,1′-biphenyl]-4-amine (TPA-DEPVI), reduces the intermolecular packing. Therefore, the side chain at N(23) and rigid frame at C(25) act as hole-trapping sites and the peripheral core blocks the electron-trapping sites. Hence, effective carrier injection and transport ability will be expected from the Cz-DPVI, Cz-DMPVI, Cz-DEPVI and TPA-DEPVI emitters. The relative carrier transport ability of the title materials was analysed using hole-only and electron-only devices. The incorporation of the side capping (*E*)-1-(2,3-dihydrobenzo[*b*][1,4]dioxin-6-yl)-2-(4-styryl)-1*H*-phenanthro[9,10-*d*]imidazole derivative at N(23) and 2-(4-(9*H*-carbazol-9-yl)phenyl)-1-methyl-1*H*-phenanthro[9,10-*d*]imidazole(Cz-DEPVI)/*N*,*N*-diphenyl-[1,1′-biphenyl]-4-amine (TPA-DEPVI) at C(25) enhanced the degree of molecular distortion and suppressed the formation of aggregation and π–π stacking in the film state, resulting in an amorphous film (smooth and pinhole-free) during fabrication.^26^ The side capping at N(23) is twisted about the 2-(4-(9*H*-carbazol-9-yl)phenyl)-1-methyl-1*H*-phenanthro[9,10-*d*]imidazole(Cz-DEPVI)/*N*,*N*-diphenyl-[1,1′-biphenyl]-4-amine (TPA-DEPVI) core with a dihedral angle of 90.1° (Cz-DPVI)/91.0° (Cz-DMPVI)/92.0° (Cz-DEPVI)/90.9° (TPA-DMPVI)/and 9-(*p*-tolyl)-9*H*-carbazole at the imidazole carbon atom (C25) is tilted about the phenanthrimidazole core at an angle of 86.1° (Cz-DPVI)/87.2° (Cz-DMPVI)/87.9° (Cz-DEPVI)/85.3° *N*,*N*-diphenyl-[1,1′-biphenyl]-4-amine (TPA-DEPVI). These orthogonal dihedral angles confirmed the non-coplanar twisting conformation of Cz-DPVI, Cz-DMPVI, Cz-DEPVI and TPA-DEPVI,^27–29^ which suppresses the red shift and harvests high quantum efficiency (*η*_ex_) in the film state by restraining intermolecular interaction.^30^ The incorporation of a highly rigid bulky moiety at C(25) and side capping at N(23) enlarged their size (*T*_d5_ and *T*_g_) and improved their thermal stability. The blue emissive phenanthroimidazole with maximum thermal stability is required for efficient devices (Table 1). The glass transition temperature (*T*_g_) of 163 °C, 174 °C, 195 °C, and 190 °C was observed for Cz-DPVI, Cz-DMPVI, Cz-DEPVI and TPA-DEPVI, respectively (Fig. 1). The side capping (*E*)-1-(2,3-dihydrobenzo[*b*][1,4]dioxin-6-yl)-2-(4-styryl)-1*H*-phenanthro[9,10-*d*]imidazole derivatives at N(23) increased the glass transition temperature compared with phenyl substitution at the imidazole nitrogen.^31^ The decomposition temperature (*T*_d5_) of compounds Cz-DPVI, Cz-DMPVI, Cz-DEPVI and TPA-DEPVI were measured to be 521 °C, 546 °C, 566 °C and 558 °C, respectively (Fig. 1). The high *T*_d5_ of all four compounds indicates the high resistance of their fused aromatic rings to thermolysis, and their high *T*_g_ and *T*_d5_ could enhance the device lifetime (Fig. 1) and expected to form a stable film during device fabrication.^32–34^ The thermal morphological stability of the Cz-DPVI, Cz-DMPVI, Cz-DEPVI and TPA-DEPVI thin films were examined *via* atomic force microscopy (AFM) measurement at room temperature and also at 100 °C for 10 h. The root-mean-square roughness (RMS) of the Cz-DPVI (0.24 nm), Cz-DMPVI (0.28 nm), Cz-DEPVI (0.32 nm) and TPA-DEPVI (0.39 nm) thin-film surfaces showed that there were no substantial changes before and after annealing (100 °C, 10 h) (Fig. 1), which also supports the suitability of these emissive materials for the fabrication of blue OLEDs.

**Table 1:** Optical and thermal properties and device performances of Cz-DPVI, Cz-DMPVI, Cz-DEPVI and TPA-DEPVI

Emitters	Cz-DPVI	Cz-DMPVI	Cz-DEPVI	TPA-DEPVI
*λ*_ab_ (nm) (sol/film)	252, 320, 356/323, 360	254, 328, 360/330, 364	260, 331, 366/334, 372	255, 332, 368/335, 370
*λ*_em_ (nm) (sol/film)	390, 408/412, 420	399, 415/420, 432	401, 428/410, 440	409, 432/415, 449
*T*_g_/*T*_d5_ (°C)	163/521	174/546	195/566	190/558
*ϕ* (soln/film)	0.76/0.83	0.80/0.89	0.82/0.91	0.78/0.86
HOMO/LUMO (eV)	−5.60/−2.58	−5.59/−2.61	−5.46/−2.70	−5.50/−2.65
*E*_g_ (eV)	3.02	2.93	2.76	2.85
*E*_S_/*E*_T_ (eV)	2.62/2.34	2.74/2.40	2.88/2.49	2.80/2.46
*τ* (ns)	1.83	1.92	2.32	2.10
*k*_r_ × 10^8^ (s^−1^)	4.15	4.17	3.53	3.71
*k*_nr_ × 10^8^ (s^−1^)	1.31	1.03	0.78	1.05
*V*_1000_ (V)	3.4	3.2	2.8	2.9
*L* (cd m^−2^)	13 629	13 841	13 955	13 856
*η*_ex_ (%)	4.6	4.9	6.9	5.7
*η*_c_ (cd A^−1^)	4.9	5.4	6.0	5.7
*η*_p_ (lm W^−1^)	4.3	5.0	5.4	5.2
CIE (*x*, *y*)	0.15, 0.08	0.15, 0.08	0.15, 0.06	0.15, 0.07
EL (nm)	419	430	439	442
*η*_IOE_ (%)^a^	22.0	23.0	24.5	23.5
*η*_S_	26.5	25.8	26.9	27.3

**Fig. 2:**
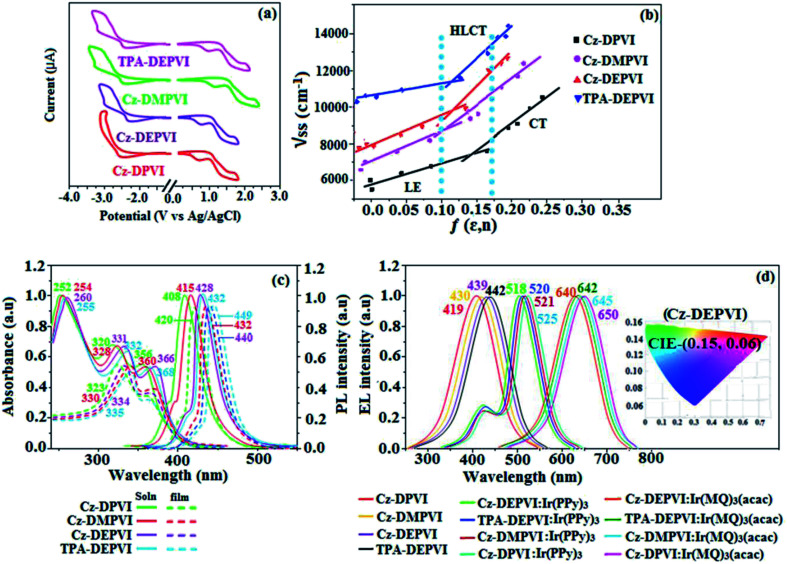
(a) Cyclic voltammograms, (b) Lippert–Mataga plot, (c) normalized absorption and emission spectra and (d) EL spectra (inset: CIE coordinates) of Cz-DPVI, Cz-DMPVI, Cz-DEPVI and TPA-DEPVI.

The HOMO orbital is distributed over the 2-(4-(9*H*-carbazol-9-yl)phenyl)-1*H*-phenanthro[9,10-*d*]imidazole fragment (Cz-DPVI, Cz-DMPVI and Cz-DEPVI)/4′-(1*H*-phenanthro[9,10-*d*]imidazol-2-yl)-*N*,*N*-diphenyl-[1,1′-biphenyl]-4-amine fragment (TPA-DEPVI); whereas, the LUMO orbital localized on the (*E*)-1-(2,3-dihydrobenzo[*b*][1,4]dioxin-6-yl)-2-(4-styryl)-1*H*-phenanthro[9,10-*d*]imidazole derivatives attached to the nitrogen atom N(23) (Fig. S2†). The HOMO and LUMO of Cz-DPVI, Cz-DMPVI, Cz-DEPVI and TPA-DEPVI display adequate separation features and their differences are quite small, which benefits the hole- and electron-transport properties (bipolar properties) and reduces the singlet–triplet splitting (Δ_ST_).^35^ Hence, the HOMO and LUMO moieties individually undertake electron and hole transport functions. The calculated electron/hole transfer integrals for Cz-DEPVI of 0.029/0.052 eV, Cz-DPVI of 0.024/0.44 eV, Cz-DMPVI of 0.26/0.50 eV and TPA-DMPVI of 0.23/0.55 eV reveal that these materials are bipolar materials. Moreover, these compounds show both reduction and oxidation waves, and thus, should have good electron and hole transport abilities (Fig. 2).

### 3.2. Photophysical properties

**Fig. 3:**
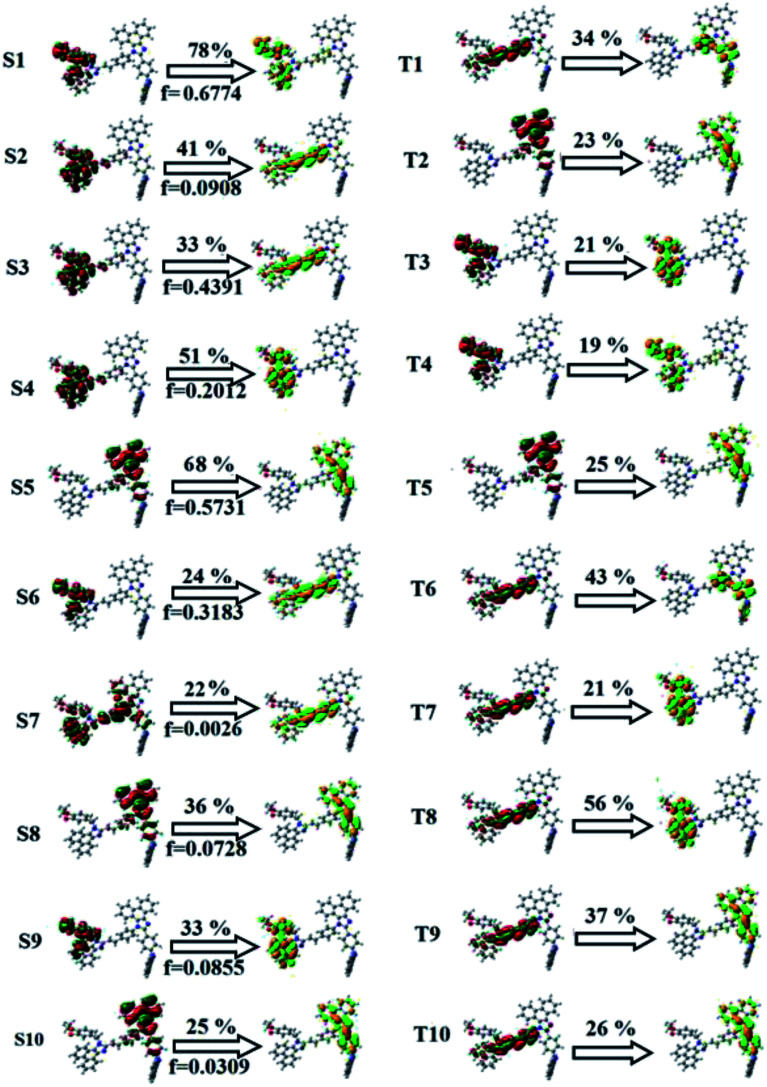
Natural transition orbital (NTO) pairs with transition character analysis for the singlet states (S_1_–S_10_) and triplet states (T_1_–T_10_) of Cz-DEPVI [*f*-oscillator strength and % weights of hole–particle].

The photophysical properties of Cz-DPVI, Cz-DMPVI, Cz-DEPVI and TPA-DEPVI were investigated *via* absorption (*λ*_abs_) and emission (*λ*_emi_) spectroscopy. The electronic spectra of Cz-DPVI, Cz-DMPVI, Cz-DEPVI and TPA-DEPVI were recorded in CH_2_Cl_2_ (Fig. 2). Since the core fragment of Cz-DPVI, Cz-DMPVI, Cz-DEPVI and TPA-DEPVI is same, these new emitters exhibit identical absorptions (solution/film) 252 (*ε*_max_ = 39 682 cm^−1^ M^−1^)/320, 356/323, 360 nm (Cz-DPVI), 254 (*ε*_max_ = 39 370 cm^−1^ M^−1^), 328, 360/330, 364 nm (Cz-DMPVI), 260 (*ε*_max_ = 38 461 cm^−1^ M^−1^), 331, 366/334, 372 nm (Cz-DEPVI) and 255 (*ε*_max_ = 39215 cm^−1^ M^−1^), 332, 368/335, 370 nm (TPA-DEPVI). The absorption in the range of 328 to 372 nm is due to intramolecular charge transfer (ICT) from the donor (carbazole/triphenylamine) to acceptor (phenanthrimidazole) and the absorption at around 248 nm is attributed to the π–π* transition.^36–38^ The intramolecular charge transfer (ICT) is further confirmed by MEP (Fig. S1†). Compared with solution, the small red shift in the corresponding films reveals that suppressed π–π* stacking exists in the solid state.^39^ The observed larger red shift supports the charge-transfer (CT) in the twisted geometry of Cz-DPVI, Cz-DMPVI, Cz-DEPVI and TPA-DEPVI. The Cz-DPVI, Cz-DMPVI, Cz-DEPVI and TPA-DEPVI derivatives show blue emission at 408, 415, 428 and 432 nm, respectively, in CH_2_Cl_2_ (Fig. 2) and their emission peak is red shifted as the polarity of the solvent increases (Fig. S3†), which is likely due to a polarization-induced spectral shift.^40^ Compared with TPA-DEPVI, Cz-DEPVI exhibits a higher blue shift in absorption and emission, which is attributed to the poor electron donor ability of Cz relative to TPA. The increased LE composition with a decrease in CT in the S_1_ emissive state is likely to be the reason for this blue shift. The full width at half maximum in the absorption spectrum of Cz-DEPVI (43 nm) is narrower than to that of TPA-DEPVI (60 nm) (Fig. 2). This observation indicates a decrease in CT component in Cz-DEPVI in the S_1_ state, which is in good agreement with the NTO description for the S_0_ → S_1_ transition. The emission peak of TPA-DEPVI and Cz-DEPVI exhibits a blue-shift relative to their parent compounds, which is contrary to the general observation, *i.e.*, the extension of π-conjugation leads to a red-shifted emission.^41^ The enhanced LE component is equivalent to the suppressed CT component in the Cz-DEPVI emissive state, which results in a blue shift. The increased LE composition is expected to result in a red-shifted PL spectrum; whereas, suppressed CT results in a blue-shifted PL spectrum. From the experimental observation it is known that the latter factor is more dominant than the former. In addition, there is an overlap between the UV and PL spectra of both TPA-DEPVI and Cz-DEPVI due to the enhanced LE character in TPA-DEPVI and Cz-DEPVI than that in their respective parent compounds. Cz-DEPVI exhibits a solvatochromic red shift (38 nm), which is smaller than that for TPA-DEPVI (68 nm) (Fig. S3, Tables S1 and S2†). Similarly, a small absorption shift of 18 nm and 27 nm was observed for Cz-DEPVI and TPA-DEPVI, respectively (Fig. S4, Tables S1 and S2†). These solvatochromic shifts confirmed that the low-lying S_1_ excited state of TPA-DEPVI and Cz-DEPVI must possess CT character.^42–44^ The % CT character of the S1 state in Cz-DEPVI is lower than that in TPA-DEPVI; whereas, the % LE character in Cz-DEPVI is higher than that in TPA-DEPVI (Table S3†). The solvatochromic effect using the Lippert–Mataga plot is displayed in Fig. 2 (Tables S1 and S2†). When the solvent polarity increased, the blue emitters exhibited a larger red shift, which supports the presence of charge transfer (CT) in these molecules.^42^ From the Stokes shift *versus* solvent polarity function plot, the ground state dipole moment (*μ*_g_) can be calculated as follows: *hc*(*υ̃*_abs_ − *υ̃*_flu_) = *hc*(*hcυ̃*^vac^_abs_ − *hcυ̃*^vac^_flu_) + 2(*μ*_e_ − *μ*_g_)^2^/ *a*_o_^3^[(*ε* – 1/2 *ε* + 1) – 1/2(*n*^2^ − 1/2*n*^2^ + 1)], where *μ*_g_ and *μ*_e_ represent the ground state and excited state dipole moment, *υ̃*_abs_ and *υ̃*^vac^_abs_ the solvent-equilibrated absorption maxima (*λ*_abs_) and that extrapolated to the gas phase, *υ̃*_flu_ and *υ̃*^vac^_flu_ the solvent-equilibrated fluorescence maxima (*λ*_emi_) and that extrapolated to the gas phase, *a*_o_ the Onsager cavity and *ε* and *n* the solvent dielectric constant and refractive index, respectively. The non-linear correlation of the Stokes shift *vs.* solvent polarity function plot revealed that there is a transformation in the fitted line between ethyl ether and methylene chloride, where the non-linear correlation supports the presence of both the locally excited state (LE) and charge transfer excited state (CT). The ground state dipole (*μ*_g_) of the blue-emitting materials, Cz-DPVI, Cz-DMPVI, Cz-DEPVI and TPA-DEPVI, was estimated from the density functional theory (DFT) calculations to be 4.8, 5.0, 5.5 and 7.02 D, respectively, which is attributed to the local exciton (LE) transition and their calculated *μ*_e_ in highly polar solvents is 22.8, 23.6, 25.8 and 30.8 D, respectively.^42^ The large *μ*_e_ in highly polar solvents (22.8, 23.6, 25.8 and 30.8 D) is close to that of the charge-transfer molecule 4-(*N*,*N*-dimethylamino)-benzonitrile (23.0 D).^44^ All these results show that CT dominates in more polar media and LE dominates in low polar media, and there is mixed contribution of LE and CT in medium polar solvents (ethyl ether and methylene chloride).^10^ The oscillator strengths of Cz-DPVI, Cz-DMPVI, Cz-DEPVI and TPA-DEPVI are displayed in Tables S4† and 2. The oscillator strength of the S_1_ state of Cz-DEPVI (0.6774, Table S4†) is higher than that of TPA-DEPVI (0.3108, Table 2), which results in higher PL efficiency (*η*_PL_) for Cz-DEPVI. Molecular modification from TPA to Cz caused an increase in the % LE in the S_1_ emissive state and enhanced the *η*_PL_ of Cz-DEPVI. To supplement the experimental results, theoretical calculations (NTO) were performed to describe the excited state characteristics of the Cz-DEPVI (Fig. 3) and TPA-DEPVI (Fig. S5†) materials.

**Table 2:** Computed [zindo (singlet or triplet, *n* states = 10)] singlet (*E*_S_) and triplet (*E*_T_) energies, oscillator strength (*f*), dipole moment (*μ*) and singlet–triplet energy difference (Δ*E*_S–T_) of TPA-DEPVI from NTOs

Energy level	*E*_S_ (eV)	Oscillator strength (*f*)	*μ* (D)	NTO Transitions	*E*_T_ (eV)	Δ*E*_S–T_ (eV)	NTO Transitions
1	0.57	0.3108	2.3747	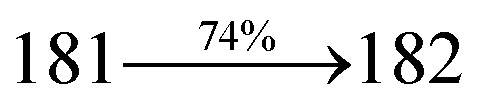	0.25	0.32	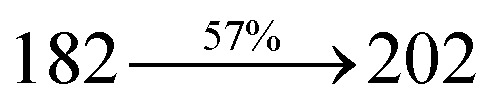
2	1.20	0.0017	0.6377	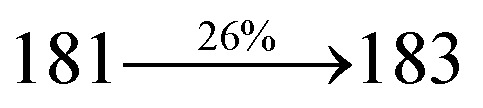	0.57	0.63	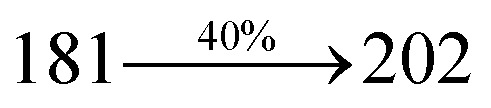
3	1.31	0.0740	0.8996	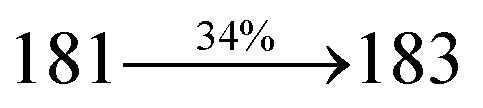	1.61	0.30	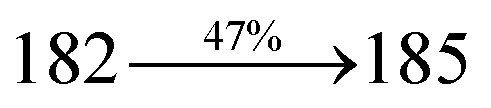
4	1.93	0.1036	2.3864	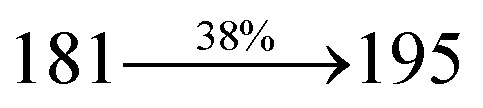	1.86	0.07	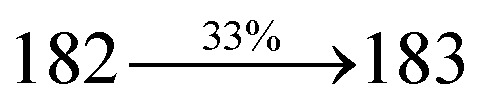
5	2.12	0.0693	1.0767	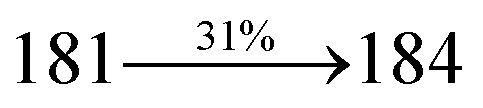	2.01	0.11	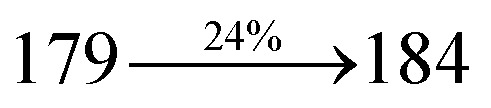
6	2.43	0.0234	1.7652	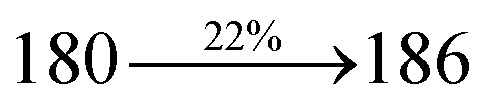	2.10	0.33	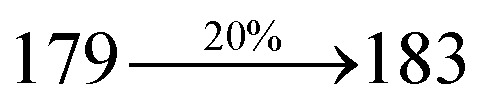
7	2.58	0.0669	0.8435	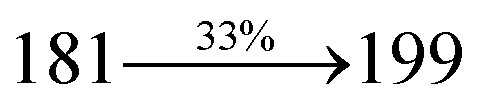	2.13	0.45	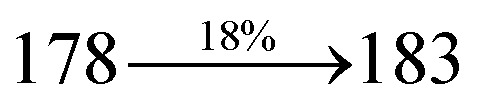
8	2.76	0.0004	0.5623	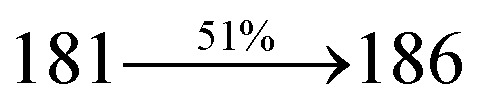	2.18	0.58	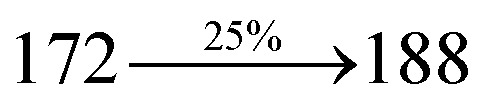
9	2.91	0.0012	1.2258	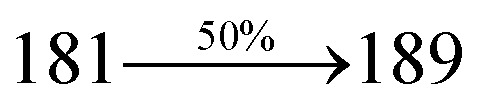	2.50	0.41	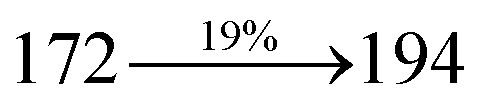
10	2.99	0.0123	1.8824	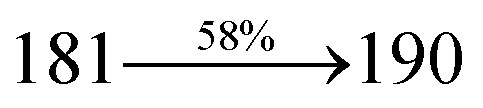	2.60	0.39	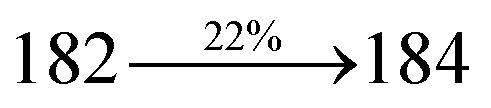

**Table 3:** Electroluminescent efficiencies of the green Cz-DPVI:Ir(PPy)_3_, Cz-DMPVI:Ir(PPy)_3_, Cz-DEPVI:Ir(PPy)_3_ and red Cz-DPVI:Ir(MQ)_3_(acac), Cz-DMPVI:Ir(MQ)_3_(acac) and Cz-DEPVI:Ir(MQ)_3_(acac)

Emitters	*V*_1000_ (V)	*L* (cd m^−2^)	*η*_ex_ (%)	*η*_c_ (cd A^−1^)	*η*_p_ (lm W^−1^)	CIE (*x*, *y*)	EL (nm)
Cz-DPVI:Ir(PPy)_3_	3.1	7146	18.2	25.3	27.0	0.32, 0.60	428, 525
Cz-DMPVI:Ir(PPy)_3_	2.8	8056	18.9	26.8	28.9	0.31, 0.60	430, 521
Cz-DEPVI:Ir(PPy)_3_	2.7	8891	19.3	27.9	33.4	0.31, 0.60	432, 518
TPA-DEPVI:Ir(PPy)_3_	2.7	8756	19.0	27.1	29.5	0.31, 0.60	431, 520
Cz-DPVI:Ir(MQ)_3_(acac)	3.0	17 215	17.6	21.1	25.4	0.64, 0.37	650
Cz-DMPVI:Ir(MQ)_3_(acac)	2.7	20 628	19.0	24.9	26.8	0.64, 0.37	645
Cz-DEPVI:Ir(MQ)_3_(acac)	2.3	40 565	19.9	26.0	30.4	0.64, 0.37	640
TPA-DEPVI:Ir(MQ)_3_(acac)	2.4	39 865	19.3	25.4	26.2	0.64, 0.37	642

**Fig. 4:**
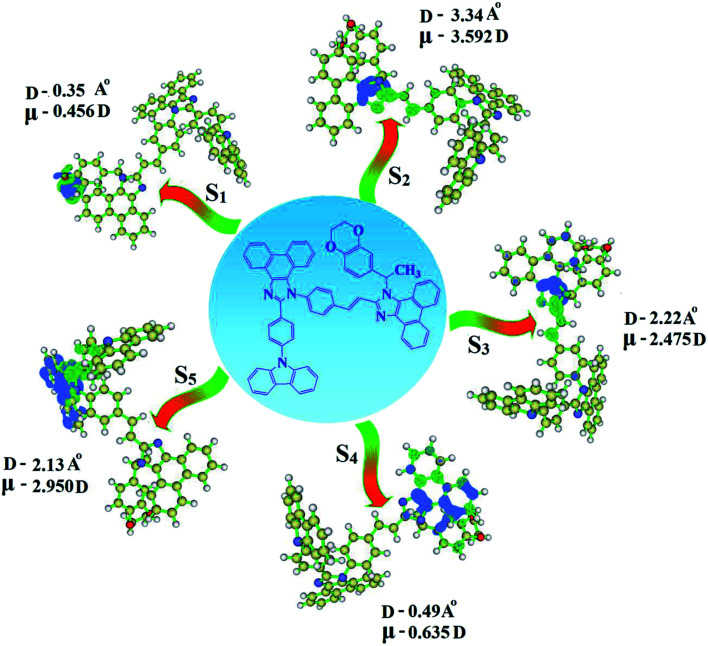
Hole and particle distribution in Cz-DEPVI [S_1_–S_5_ states, green increasing electron density and blue decreasing electron density (density = transition = *n* IOp (6/8 = 3))].

The overlap density between the hole and particle depends on the configuration of the donor–acceptor architecture and the magnitude of the overlap intensity tunes the % LE and % CT in the S_1_ state (Fig. S6:† TPA-DEPVI and Fig. 4: Cz-DEPVI).

**Fig. 5:**
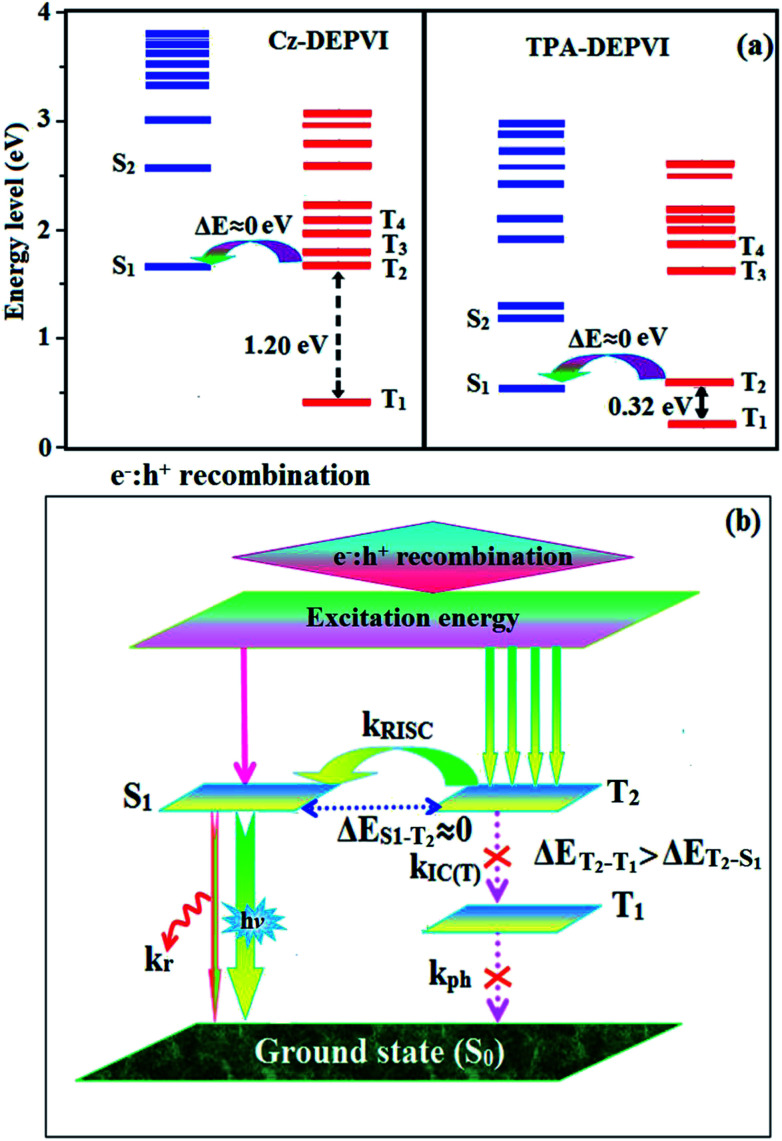
(a) Energy level of the singlet (S) and triplet (T) states of Cz-DEPVI and TPA-DEPVI and (b) schematic of the exciton decay process after hole and electron recombination in OLEDs.

The NTO of the S_1_ and S_2_ excited states of Cz-DEPVI and TPA-DEPVI exhibit a hybrid splitting state character, which is derived from the interstate coupling of the LE and CT levels (Tables S4† and 2). The hole contours on the Cz and TPA moieties are in the opposite phase between the S_1_ and S_2_ states; whereas, the particle on the phenanthrimidazole moiety is same between the S_1_ and S_2_ states for Cz-DEPVI and TPA-DEPVI, respectively. This implies that the interstate hybridization coupling occurs through the positive and negative linear combination between the LE and CT state wavefunction: *Ψ*_S_1_/S_2__ = *c*_LE_*Ψ*_LE_ ± *c*_CT_*Ψ*_CT_. The percentage of pure CT level of Cz-DEPVI is less than that of TPA-DEPVI as a result of the weaker donor ability of Cz than TPA, leading to the LE-dominated S_1_ state in Cz-DEPVI (LE ∼ 30%) and LE/CT-balanced S_1_ state in TPA-DEPVI (LE ∼ 20%). As a result, Cz-DEPVI should exhibit higher photoluminance efficiency (*η*_PL_) and a blue-shifted emission relative to TPA-DEPVI. The singlet state energies were estimated from the geometry of the S_0_ state. The excited state singlet and triplet energies were calculated using the fluorescence and phosphorescence spectra of Cz-DEPVI and TPA-DEPVI at low temperature. The maximum absorption at low temperature was 335 nm for Cz-DEPVI and 444.1 nm for TPA-DEPVI and isoenergetic vibronic emission maximum absorption was 336 nm for Cz-DEPVI and 446.0 nm for TPA-DEPVI) in the low-temperature phosphorescence spectrum. A large energy gap occurred between T_1_ and T_2_ for both Cz-DEPVI (1.21 eV) and TPA-DEPVI (0.32 eV), originating from their same phenanthrimidazole acceptor group, and the energy gap between T_1_ and T_2_ for Cz-DEPVI is larger than that for TPA-DEPVI (Fig. 5).^45,46^ A very small Δ*E*_ST_ ≈ 0 was observed between the S_1_ and T_2_ states, facilitating the RISC (T_2_ → S_1_) process in both Cz-DEPVI (Table S5†) and TPA-DEPVI (Table S6†) as a result of their HLCT state character (Fig. 5). Thus, compared with TPA-DEPVI, Cz-DEPVI can be expected to exhibit high photoluminance efficiency (*η*_PL_) and high exciton utilisation efficiency (*η*_S_) and further enhance the external quantum efficiency (*η*_EQE_) of fluorescent OLEDs as a result of the increased LE component in its S_1_ state. The formation of a single emissive state can be analysed though the excitation energies of the LE and CT states (Tables S5 and S6†).

**Fig. 6:**
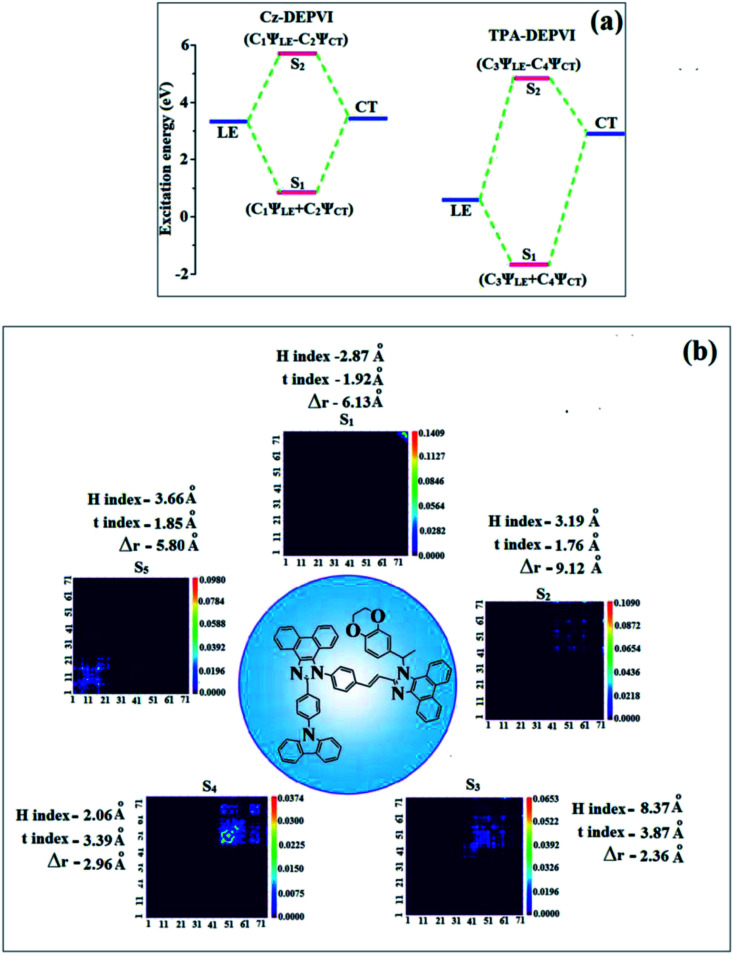
(a) Schematic diagram of the hybridization processes of the LE and CT states of Cz-DEPVI and TPA-DEPVI and (b) computed contour plots of the transition density matrices (TDM) of Cz-DEPVI for [S_1_–S_5_ states: density = transition = *n*/IOp(6/8 = 3)].

A similar hole–electron wavefunction between S_1_ and S_2_ was observed in both Cz-DEPVI and TPA-DEPVI, which indicates a quasi-equivalent hybridization between the LE and CT states as a result of their almost isoenergies for the initial LE and CT states (Fig. 6). Therefore, the degree of hybridization between the LE and CT states depends not only on the initial *E*_LE_ − *E*_CT_ energy gap but also on their interstate coupling strength.^47^ Compared with non-equivalent hybridization, quasi-equivalent hybridization is expected to achieve the combination of high *η*_PL_ and high *η*_S_ to maximize the EL efficiency of fluorescent OLED materials due to more balanced LE and CT components in the HLCT state of Cz-DEPVI and TPA-DEPVI. In Cz-DEPVI and TPA-DEPVI, the LE state is more stabilized than the CT state and the energy gap (*E*_S_2__ − *E*_S_1__) is small when compared with that of their parent compounds, which result in quasi hybridization. In the case of Cz-DEPVI, the energy gap (*E*_S_2__ − *E*_S_1__) is reduced, which results in effective hybridization and improves the OLED efficiency. The composition of the HLCT can be discussed using the wavefunction of the electron–hole pair transition density matrix (TDM), which was plotted in a two-dimension color-filled map (Fig. 6: Cz-DEPVI and Fig. S7: TPA-DEPVI†). The axes represent the atom in a molecule, which is proportional to the probability of finding an electron and hole in the atomic orbital located on a non-hydrogen atom. Specifically, the diagonal represents the LE component localized on the main backbone, while the off-diagonal region shows the CT component. The qualitatively calculated percentages of LE and CT in the S_1_–S_10_ and T_1_–T_10_ states are displayed in Table S3.† This also supports that HLCT state contributes to hybridization besides the LE and CT states. Upon excitation, an electron is transferred from the donor and localized on the acceptor. Depending on the intramolecular geometrical and electronic coupling, the transferred electron is delocalized from the region of the nearby donor molecule to the vicinity of the acceptor. This effect can be qualitatively studied by analyzing the electron density distribution in the ground and excited states. The computed electron–hole properties, distance between hole and electron, transition density, *H* and *t* indies and RMSD of an electron and hole for Cz-DEPVI and TPA-DEPVI are displayed in Tables S7 and S8.†

**Fig. 7:**
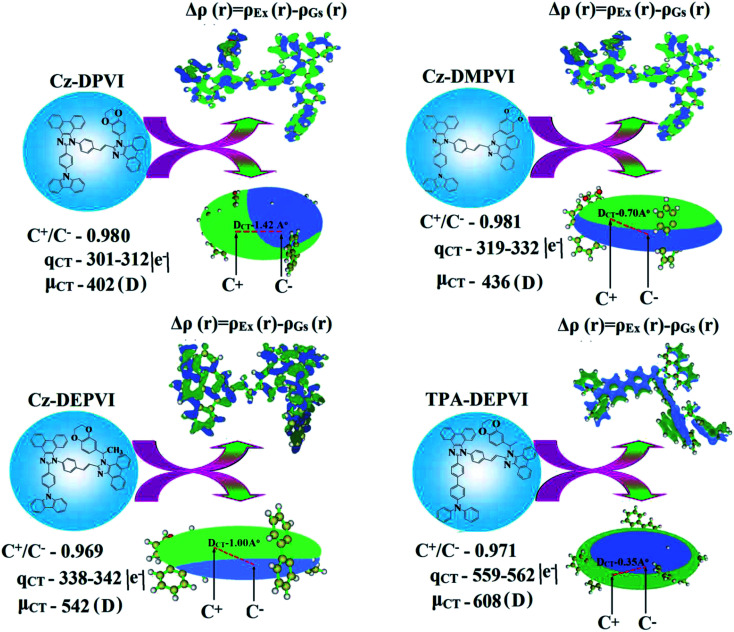
Computed difference in total density for the ground and excited states [Δ*ρ*(*r*) = *ρ*_Ex_(*r*) − *ρ*_Gs_(*r*); isosurface for Cz-DPVI, Cz-DMPVI, Cz-DEPVI, (0.0000006 a.u.) and TPA-DEPVI (0.15 a.u.)] and graphical representation of the *D*_CT_ and centroid of charges [*C*+(*r*)/*C*−(*r*); isosurface for Cz-DEPVI (0.29 a.u.) and TPA-DEPVI (0.1 a.u.)].

The integral value for the holes and electrons in Cz-DEPVI (Table S9†) is less than that for TPA-DEPVI (Table S10†) with the transition density. The integral overlap of the hole–electron distribution (*S*) is a measure of the spatial separation of holes and electrons. The integral overlap (*S*) of holes and electrons and the distance (*D*) between the centroids of the holes and electrons evidence the existence of LE and CT states (Tables S9 and S10†). Compared with their parent compounds, Cz-DEPVI and TPA-DEPVI have small *D* and high *S* values; however, the small *D* and high *S* of TPA-DEPVI in comparison with Cz-DEPVI indicate the charge transfer (CT) is higher for the TPA-DEPVI isomer. The variation in dipole moment with respect to S_0_ state was also plotted, which was directly evaluated based on the position of the centroid of the holes and electrons. The RMSD of the holes and electrons characterizes the extent of their distribution, were the RMSD of both electrons and holes in Cz-DEPVI (Table S7†) is higher in the *X* direction, which indicates the electron and hole distribution is much broader in the *X* direction. In contrast, the RMSD of the electrons in TPA-DEPVI (Table S8†) is smaller and that of the holes is higher than Cz-DEPVI. The *H* index (half the sum of the axis of the anisotropic density variation distribution) measures the spread of the positive and negative regions related to CT. The CT index, *i.e.*, the *t* index difference between *D*_CT_ and the *H* index, is another measure of the hole–electron separation (eqn (S15) and (S16)†). The *D*_CT_ of Cz-DPVI, Cz-DMPVI, Cz-DEPVI and TPA-DEPVI was calculated to be 1.422, 0.703, 1.008 and 0.352, respectively (Fig. 7 and Table S11†). For both Cz-DEPVI and TPA-DEPVI, the non-zero *t* is negative in all directions, where the overlap of holes and electrons is very severe (Fig. 7, Table S11†) and the eigenvalue is greater than 0.96, which support the hybridization and is described in terms of dominant excitation pair as 94% transition.

This is further evidenced by the Δ*r* index (Tables S5 and S6†). The Δ*r* index (eqn (S1)†) is the average hole (h^+^)–electron (e^−^) distance (*d*_h^+^–e^−^_) upon excitation, which indicates the nature of the excitation type, LE or CT. Specifically, valence excitation (LE) is related to short distances (*d*_h^+^–e^−^_), while larger distances (*d*_h^+^–e^−^_) are related to CT excitation. The triplet exciton is transformed into singlet excitons in TPA-DEPVI and Cz-DEPVI *via* the RISC process with a high energy excited state (hot CT channel),^13,48^ which is beneficial for triplet exciton conversion in the electroluminescence process without any delayed fluorescence. The CT excitons are formed with a weak binding energy (*E*_b_) on higher excited states.^49^ As a result, the exciton utilization (*η*_S_) can be harvested in TPA-DEPVI and Cz-DEPVI similarly to phosphorescent materials. The quasi-equivalent hybridized materials TPA-DEPVI and Cz-DEPVI exhibit excellent device performances due to the fine modulation in their excited states, where the enhanced LE component and hybridization between the LE and CT components result in high *η*_PL_ and high *η*_S_. Thus, the coexisting LE/CT composition in TPA-DEPVI and Cz-DEPVI harvested high *η*_PL_ and high *η*_S_ and enhanced the OLED performances (Table 1). These compounds exhibit blue emission with high quantum yields (solution/film), *i.e.*, Cz-DPVI (0.76/0.83), Cz-DMPVI (0.80/0.89), Cz-DEPVI (0.82/0.91) and TPA-DEPVI (0.78/0.86) and high fluorescence efficiencies, which are essential for efficient blue OLEDs. The triplet energy (*E*_T_) levels were estimated to be 2.34 eV (Cz-DPVI), 2.40 eV (Cz-DMPVI), 2.49 eV (Cz-DEPVI) and 2.46 eV (TPA-DEPVI), which are sufficiently high for the excitation of green and red phosphorescent dopants.^22^ The Δ*E*_ST_ of these hosts are relatively small, which is due to their spatially separated HOMO and LUMO levels. The small Δ*E*_ST_ is advantageous for efficient energy transfer from the triplet excited state of the hosts to green and red phosphorescent emitters.^27^

**Fig. 8:**
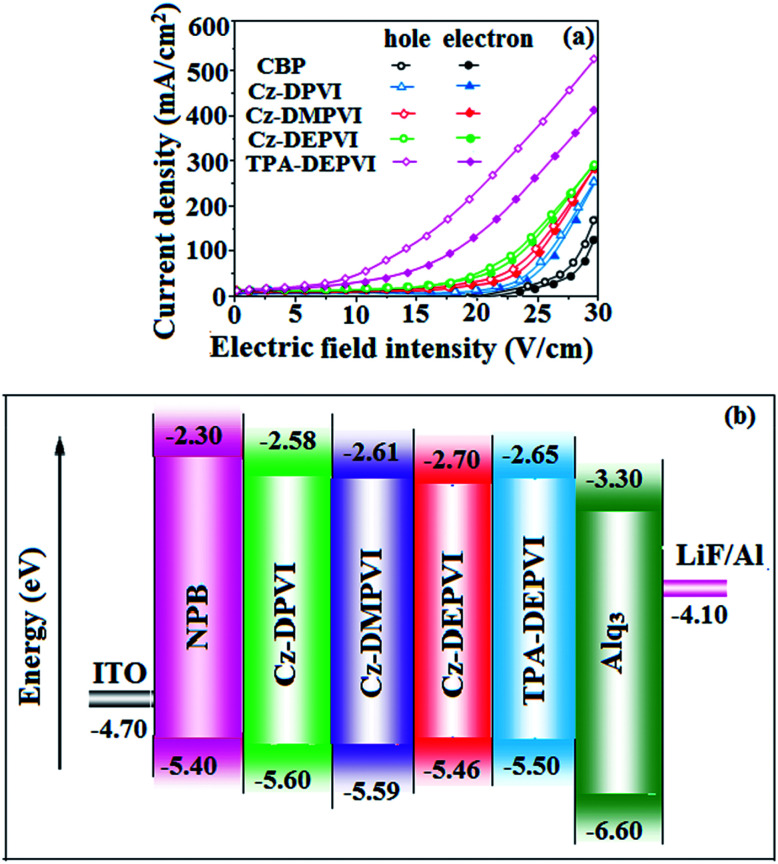
(a) Hole-only and electron-only devices based on Cz-DPVI, Cz-DMPVI, Cz-DEPVI and TPA-DEPVI and (b) energy level diagram of the non-doped devices.

To evaluate the carrier injection and transport properties of the materials, hole-only and electron-only devices were fabricated as follows: (a) ITO/HATCN(10 nm)/Cz-DPVI/Cz-DMPVI/Cz-DEPVI (60 nm)/HATCN (10 nm)/LiF (1 nm)/Al (100 nm) (hole-only device IV) and (b) ITO/TPBi (10 nm)/Cz-DPVI/Cz-DMPVI/Cz-DEPVI (60 nm)/TPBi (10 nm)/LiF (1 nm)/Al (100 nm) (electron-only device V). Fig. 8 shows the current density *versus* voltage characteristics of the hole-only and electron-only devices. The electron current density of the Cz-DPVI-, Cz-DMPVI-, Cz-DEPVI- and TPA-DEPVI-based devices is higher than that for the CBP-based device. This reveals that these materials have better electron injection and transport properties than CBP. The difference in current density between the hole-only and electron-only devices based on Cz-DPVI, Cz-DMPVI, Cz-DEPVI and TPA-DEPVI is much smaller than that based on CBP at the same voltage, which suggests that these materials are potential bipolar materials capable of transporting electrons and holes in devices.^50–52^

**Fig. 9:**
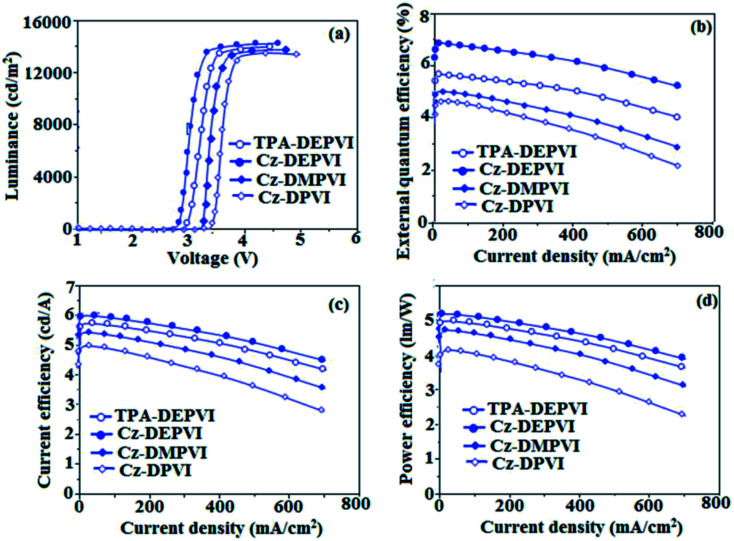
Electroluminescence performances: (a) luminance *versus* voltage, (b) external quantum efficiency *versus* current density, (c) current efficiency *versus* current density and (d) power efficiency *versus* current density of Cz-DPVI, Cz-DMPVI, Cz-DEPVI and TPA-DEPVI.

The observed intense blue emission and high *T*_g_ of Cz-DPVI, Cz-DMPVI, Cz-DEPVI and TPA-DEPVI support their suitability to serve as blue emitters in OLEDs. The device performances of the blue emitters were analysed by fabricating non-doped OLEDs with the configuration of ITO/NPB (1,4-bis(1-naphthylphenylamino)-biphenyl) (50 nm)/Cz-DPVI/Cz-DMPVI/Cz-DEPVI/TPA-DEPVI (30 nm)/BCP (2,9-dimethyl-4,7-diphenyl-1,10-phenanthroline) (15 nm)/Alq_3_(tris-(8-hydroxyquinoline) aluminum) (50 nm)/LiF (1 nm)/Al (100 nm) (Fig. 8). The device performances are displayed in Table 1. It is clear from Fig. 9 that the three new Cz-DPVI-, Cz-DMPVI-, Cz-DEPVI- and TPA-DEPVI-based devices exhibit high brightness at low voltage.

The EL spectra of the devices are similar to the PL spectra of Cz-DPVI, Cz-DMPVI, Cz-DEPVI and TPA-DEPVI in the film state (Fig. 2). The hole injection barrier between Cz-DEPVI and the hole transport layer is very small, and thus results in effective electron–hole radiative recombination in the emissive layer. The small injection barrier of Cz-DPVI (0.20 eV), Cz-DMPVI (0.19 eV), Cz-DEPVI (0.06 eV) and TPA-DEPVI (0.10 eV) may account for their observed low turn-on voltages [Cz-DPVI (3.4 V), Cz-DMPVI (3.2 V), Cz-DEPVI (2.8 V) and TPA-DEPVI (2.9 V)]. The EQE of OLEDs can be calculated as follows: EQE = *η*_out_ × *η*_rc_ × *η*_γ_ × *Φ*_PL_,^53^ where, *η*_out_ is the light outcoupling efficiency (20%), *η*_rc_ the product of the charge recombination efficiency (100%), *η*_γ_ the efficiency of radiative exciton production (25%) and *Φ*_PL_ the photoluminescence quantum yield of the emitters. The *η*_r_ for the Cz-DPVI-, Cz-DMPVI-, Cz-DEPVI- and TPA-DEPVI-based devices was calculated to be in the range of 20–27%, which indicates *γ* is less than 100% due to very small unbalanced carrier transportation.^34^ The maximum external quantum efficiency (*η*_ex_) and current efficiency (*η*_c_) of the Cz-DPVI-, Cz-DMPVI-, Cz-DEPVI- and TPA-DEPVI-based devices are 4.4%, 4.6%, 4.9% and 4.7% and 4.9, 5.4, 6.0 and 5.7 cd A^−1^, respectively. This result can be attributed to the more balanced charge-transporting properties within the emissive layer, which are achieved by the better charge injection provided by the hole transport layer. The Cz-DPVI-, Cz-DMPVI-, Cz-DEPVI- and TPA-DEPVI-based devices showed high power efficiencies (*η*_p_) of 4.3 (3.4 V), 5.0 (3.2 V), 5.4 (2.8 V) and 5.2 (2.9 V) lm W^−1^ with CIE coordinates of (0.15, 0.08), (0.15, 0.08), (0.15, 0.06) and (0.15, 0.07), respectively. The internal quantum efficiencies (*η*_IQE_) of Cz-DPVI, Cz-DMPVI, Cz-DEPVI and TPA-DEPVI were calculated to be 22.0%, 23.0%, 24.5% and 23.5%, and their exciton utilization efficiencies (*η*_S_) 26.5%, 25.8%, 26.9% and 27.3%, respectively. The high *η*_S_ of the Cz-DPVI-, Cz-DMPVI-, Cz-DEPVI- and TPA-DEPVI-based devices is due to several different mechanisms, such as TADF, TTA and HLCT. Further, the luminance *versus* current density plot exhibits a linear relationship, and hence the high *η*_S_ cannot be attributed to the TTA process. The devices with Cz-DPVI, Cz-DMPVI, Cz-DEPVI and TPA-DEPVI showed the maximum luminance (*L*) of 13 629, 13 841, 13 955 and 13 856 cd m^−2^, respectively. These experimental results demonstrate clearly that the additional triplet excitons were utilized in the OLED applications due to the HLCT character of Cz-DPVI, Cz-DMPVI, Cz-DEPVI and TPA-DEPVI, as shown in Scheme 1, and shows the accuracy of our molecular design strategy. The EL brightness exhibited a linear relationship with current density for these compounds, indicating that the contribution from triplet–triplet annihilation was insignificant.^54^ The lifetime measurement revealed that this intercrossed excited state in different polar solvents should be a hybridized local and charge transfer state instead of a two-species state through the simple addition of LE and CT. The mono-exponential lifetime [1.83 ns (Cz-DPVI), 1.92 ns (Cz-DMPVI), 2.32 ns (Cz-DEPVI), and 2.10 ns (TPA-DEPVI)] demonstrates that the intercrossed LE and CT in the moderate polarity solvent formed one hybridized HLCT state, which supports the molecular design (Fig. 2). Since no delayed fluorescence was recorded from transient PL, the high EQEs did not seem to be in accordance with TADF.^54^ The emission wavelength of both Cz-DEPVI and TPA-DEPVI in film is close to that in ethyl ether, which supports the HLCT state formed in the Cz-DEPVI and TPA-DEPVI films. The radiative transition rate (*k*_r_) and the non-radiative transition rate (*k*_nr_) of Cz-DEPVI and TPA-DEPVI were calculated using their lifetime and quantum yield values. The radiative (*k*_r_ = *ϕ*/*τ*)/non-radiative (*k*_nr_ = 1/*τ* − (*ϕ*/*τ*)) (*k*_r_/*k*_nr_ 10^8^ s^−1^) rate constants of Cz-DPVI (4.15/1.31), Cz-DMPVI (4.17/1.03), Cz-DEPVI (3.53/0.78) and TPA-DEPVI (3.71/1.05) (Table 1) reveal that *k*_r_ > *k*_nr_. Compared with TPA-DEPVI, the *k*_r_ of Cz-DEPVI increased and the *k*_nr_ of Cz-DEPVI decreased. This result is also in good agreement with the aim of our molecular design.

The current and power efficiencies of the devices based on Cz-DEPVI (6.0 cd A^−1^ and 5.4 lm W^−1^) and TPA-DEPVI (5.7 cd A^−1^ and 5.2 lm W^−1^) are higher than that of the devices based on TPA-PA (1.16 cd A^−1^ and 0.65 lm W^−1^), TPA-NzP (1.00 cd A^−1^ and 0.77 lm W^−1^)^55^ and *m*TPA-PPI (0.84 cd A^−1^ and 0.48 lm W^−1^), respectively. Also the external quantum yield of Cz-DEPVI (82%) and Cz-DEPVI (78%) is higher than that of (i) Cz-BzP (69.7%) and TPA-BzP (49.2%)^56^ (ii) CBI (21%) and MCB (24%) and (iii) PPI-pCNCz (54%). Wang *et al.*^57^ reported that the thickness of the LBPPI emissive layer alters the current efficiency (50 nm: 0.01 cd A^−1^, 40 nm: 0.13 cd A^−1^, 30 nm: 0.40 cd A^−1^ and 20 nm: 0.68 cd A^−1^). The current efficiency obtained in the present study with a thickness of 30 nm for Cz-DEPVI (6.0 cd A^−1^) and 30 nm for TPA-DEPVI (5.7 cd A^−1^) is higher than that previously reported.^55–57^ Hence, it is possible to improve the efficiency of these materials through modification of the thickness of the emissive layer. Accordingly, efforts will be made to modify the thickness of the emissive layer to enhance the efficiency and to increase the radiative rate in our future studies. The weak donor carbazole-substituted phenanthrimidazole exhibits a current efficiency of 0.88 cd A^−1^ and power efficiency of 0.30 lm W^−1^ and Gao *et al.* reported a carbazole-substituted compound with a current efficiency of 0.65 cd A^−1^ and power efficiency of 0.48 lm W^−1^.^57^ These carbazole-substituted compounds exhibited lower efficiencies and power efficiencies than that obtained in our studies with Cz-DPVI (4.9 cd A^−1^ and 4.3 lm W^−1^), Cz-DMPVI (5.4 cd A^−1^ and 5.0 lm W^−1^) and Cz-DEPVI (6.0 cd A^−1^ and 5.4 lm W^−1^), respectively. The device efficiency indicates that Cz-DPVI, Cz-DMPVI, Cz-DEPVI and TPA-DEPVI are excellent fluorescent OLED materials.

**Fig. 10:**
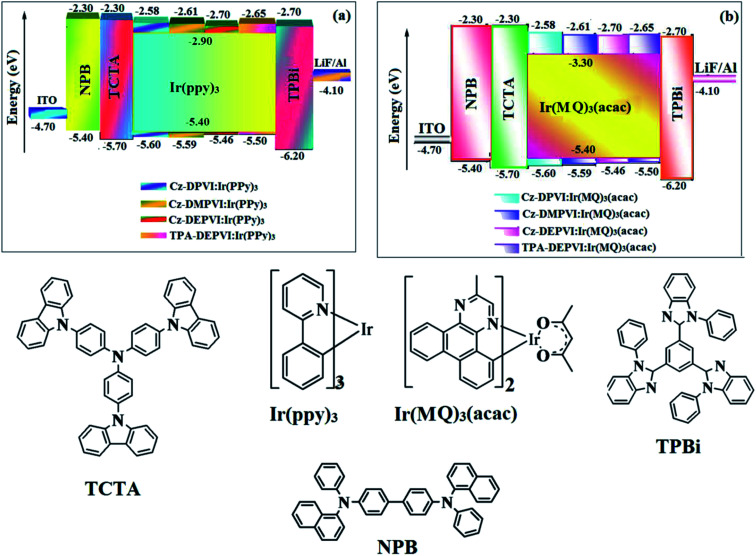
Energy level diagrams of the green (a) and red (b) devices with the molecular structures of the functional materials used in the devices.

Cz-DPVI, Cz-DMPVI, Cz-DEPVI and TPA-DEPVI were also employed as host materials for green and red phosphorescent dopants. The fabricated green and red devices had the configuration of ITO/NPB (40 nm)/TCTA (5 nm)/Cz-DPVI (30 nm): 5 wt% Ir(ppy)_3_/Cz-DMPVI (30 nm): 5 wt% Ir(ppy)_3_/Cz-DEPVI (30 nm): 5 wt% Ir(ppy)_3_/TPA-DEPVI (30 nm): 5 wt% Ir(ppy)_3_TPBI (50 nm)/LiF (1 nm)/Al (100 nm)]: ITO/NPB (40 nm)/TCTA (5 nm)/Cz-DPVI (30 nm): 8 wt% Ir(MQ)_2_(acac)/Cz-DMPVI (30 nm): 8 wt% Ir(MQ)_2_(acac)/Cz-DEPVI (30 nm): 8 wt% Ir(MQ)_2_(acac)/TPA-DEPVI (30 nm): 8 wt% Ir(MQ)_2_(acac)/TPBI (50 nm)/LiF (1 nm)/Al (100 nm)], respectively (Fig. 10), and Ir(ppy)_3_-*fac*-tris(2-phenylpyridine) iridium(iii) and Ir(MQ)_2_(acac)-bis(2-methyldibenzo-[*f*,*h*] quinoxaline) acetylacetonate iridium(iii) were used as emissive layers for the green and red devices, respectively.

**Fig. 11:**
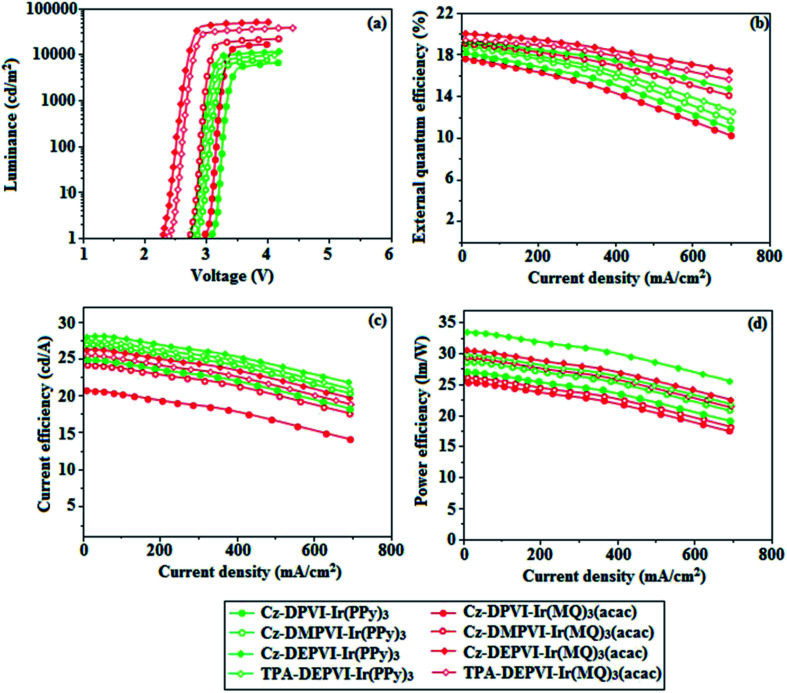
Device efficiencies: (a) Luminance *versus* voltage, (b) external quantum efficiency *versus* current density, (c) current efficiency *versus* current density and (d) power efficiency *versus* current density of the green devices based on Cz-DPVI:Ir(PPy)_3_, Cz-DMPVI:Ir(PPy)_3_, Cz-DEPVI:Ir(PPy)_3_ and TPA-DEPVI:Ir(PPy)_3_ and red devices based on Cz-DPVI:Ir(MQ)_3_(acac), Cz-DMPVI:Ir(MQ)_3_(acac), Cz-DEPVI:Ir(MQ)_3_(acac) and TPA-DEPVI:Ir(MQ)_3_(acac).

The device performances are shown in Fig. 11. The EL spectra are similar to the PL spectra of the doped thin films (Fig. 2). The green device based on Cz-DEPVI (30 nm): 5 wt% Ir(ppy)_3_ exhibited the maximum luminance of 8891 cd m^−2^, and maximum current and power efficiencies of 27.9 cd A^−1^ and 33.4 lm W^−1^, respectively at 2.7 V (Table 3). The maximum external quantum efficiencies of the devices based on Cz-DPVI:Ir(ppy)_3_, Cz-DMPVI:Ir(ppy)_3_, Cz-DEPVI:Ir(ppy)_3_ and TPA-DEPVI:Ir(ppy)_3_ are 18.2%, 18.9%, 19.3% and 19.0%, respectively. Similar to the green devices, red device based on Cz-DEPVI:Ir(MQ)_2_(acac) exhibited the maximum luminance of 40 565 cd m^−2^ and excellent EL efficiencies (*η*_ex_: 19.9%, *η*_c_: 26.0 cd A^−1^, and *η*_p_: 30.4 lm W^−1^) with CIE coordinates of (0.64, 0.37) among the red devices, including Cz-DPVI:Ir(MQ)_2_(acac) (*L*: 17 215 cd m^−2^, *η*_ex_: 17.6%, *η*_c_: 21.1 cd A^−1^, and *η*_p_: 25.4 lm W^−1^ with CIE coordinates of (0.64, 0.37)), Cz-DMPVI:Ir(MQ)_2_(acac) (*L*: 20 628 cd m^−2^, *η*_ex_: 19.0%, *η*_c_: 24.9 cd A^−1^, and *η*_p_: 26.8 lm W^−1^ with CIE coordinates of (0.64, 0.37)) and TPA-DEPVI:Ir(MQ)_2_(acac) (*L*: 39 865; cd m^−2^, *η*_ex_: 19.3%, *η*_c_: 25.4 cd A^−1^, *η*_p_: 26.2 lm W^−1^ with CIE coordinates of (0.64, 0.37)). The above experimental results demonstrate that Cz-DPVI, Cz-DMPVI, Cz-DEPVI and TPA-DEPVI are universal host materials for green and red phosphorescent emitters (Table 3).

## 4. Conclusion

We reported efficient new deep blue-emitting materials Cz-DPVI, Cz-DMPVI, Cz-DEPVI and TPA-DEPVI with the D–A geometry, which exhibit dual charge transport properties and show excellent thermal properties with high glass-transition temperature. The photophysical properties, film morphology, and electrochemical and electroluminescent properties of Cz-DEPVI and TPA-DEPVI can be tuned by chemical modification from Cz-DEPVI to TPA-DEPVI by changing the strong donor TPA moiety with the weak donor Cz moiety. The deep blue emission with carrier transport abilities of Cz-DPVI, Cz-DMPVI, Cz-DEPVI and TPA-DEPVI reveal that the non-doped devices with these compounds exhibit maximum external quantum efficiencies, and current and power efficiencies, *i.e.*, Cz-DPVI (4.4%, 4.9 cd A^−1^ and 4.3 lm W^−1^), Cz-DMPVI (4.6%, 5.4 cd A^−1^ and 5.0 lm W^−1^), Cz-DEPVI (4.9%, 6.0 cd A^−1^ and 5.4 lm W^−1^) and TPA-DEPVI (4.7%, 5.7 cd A^−1^ and 5.2 lm W^−1^), respectively. These materials exhibit blue emission with CIE coordinates of (0.15, 0.08) for Cz-DPVI, (0.15, 0.08) for Cz-DMPVI, (0.15, 0.06) for Cz-DEPVI and (0.15, 0.07) for TPA-DEPVI at a low driving voltage. These blue emissive materials with good carrier transport properties were also employed as hosts for green and red phosphorescent devices. The maximum external quantum efficiencies of the devices based on Cz-DPVI:Ir(ppy)_3_, Cz-DMPVI:Ir(ppy)_3_, Cz-DEPVI:Ir(ppy)_3_ and TPA-DEPVI:Ir(ppy)_3_ were 18.2%, 18.9%, 19.3% and 19.0%, respectively. Similarly to the green devices, the red device based on Cz-DEPVI:Ir(MQ)_2_(acac) exhibited the maximum luminance of 40 565 cd m^−2^ and excellent EL efficiencies (*η*_ex_: 19.9%, *η*_c_: 26.0 cd A^−1^, *η*_p_: 30.4 lm W^−1^) with CIE coordinates of (0.64, 0.37). The present findings demonstrate a new route to harvest high-performance full-color OLEDs by utilizing bipolar luminescent materials possessing the D–A molecular structure. Also, the crucial role of host materials with high triplet energy and the small Δ_ST_ strategy can be used for the development of efficient green and red OLEDs.

## Conflicts of interest

There are no conflicts to declare.

## Supplementary Material

RA-009-C8RA09486A-s001
