# Supplementary material for: Efficient full-colour organic light-emitting diodes based on donor–acceptor electroluminescent materials with a reduced singlet–triplet splitting energy gap
Source: RSC Adv. 2019 Jan 23;9(6):2948–66. doi: 10.1039/c8ra09486a (PMC9060244; doi:10.1039/c8ra09486a)
Supplement: RA-009-C8RA09486A-s001 [file RA-009-C8RA09486A-s001.pdf]

Electronic Supplementary Material (ESI) for RSC Advances.
This journal is © The Royal Society of Chemistry 2019

Supporting information

Efficient full colour organic light emitting diodes based on donor–acceptor electroluminescent materials with reduced singlet–triplet splitting energy gap

Jayaraman Jayabharathi*, Ramaiyan Ramya, Venugopal Thanikachalam, Palanivel Jeeva, Elayaperumal Sarojpurani[a]

Department of Chemistry, Annamalai University, Annamalai nagar, Tamilnadu- 608 002, India
[a]Sri Manakula Vinayagar Engineering College

* Address for correspondence

Dr. J. Jayabharathi
Professor of Chemistry
Department of Chemistry
Annamalai University
Annamalai nagar 608 002
Tamilnadu, India.
Tel: +91 9443940735
E-mail: jtchalam2005@yahoo.co.in

Contents

SI–I: Charge-Transfer intexes

SI–VII: Figures

SI–XI: Tables

SI–I: Charge–Transfer intexes

The hole–particle pair interactions have been related to the distance covered during the excitations one possible descriptor Δr intex could be used to calculate the average distance which is weighted in function of the excitation coefficients.

$$\Delta r = \frac{\Sigma_{ia} k_{ia}^{2} \left| \langle \varphi_a | r | \varphi_a \rangle - \langle \varphi_i | r | \varphi_i \rangle \right|}{\Sigma_{ia} K_{ia}^{2}} \quad \text{.................. (S1)}$$

where $|\langle \varphi_i | r | \varphi_i \rangle|$ is the norm of the orbital centroid [1–4]. Δr–index will be expressed in Å. The density variation associated to the electronic transition is given by

$$\Delta\rho(r) = \rho_{EX}(r) - \rho_{GS}(r) \quad \text{...................... (S2)}$$

where $\rho_{GS}(r)$ and $\rho_{EX}(r)$ are the electronic densities of to the ground and excited states, respectively. Two functions, $\rho_+(r)$ and $\rho_-(r)$, corresponds to the points in space where an increment or a depletion of the density upon absorption is produced and they can be defined as follows:

$$\rho_+(r) = \begin{cases} \Delta\rho(r) & if\ \Delta\rho(r) > 0 \\ 0 & if\ \Delta\rho(r) < 0 \end{cases} \quad \text{........................ (S3)}$$

$$\rho_-(r) = \begin{cases} \Delta\rho(r) & if\ \Delta\rho(r) < 0 \\ 0 & if\ \Delta\rho(r) > 0 \end{cases} \quad \text{........................ (S4)}$$

The barycenters of the spatial regions R_+ and R_- are related with $\rho_+(r)$ and $\rho_-(r)$ and are shown as

$$R_+ = \frac{\int r\rho_+(r)dr}{\int \rho_+(r)dr} = (x_+, y_+, z_+) \quad \text{..................... (S5)}$$

$$R_- = \frac{\int r\rho_-(r)dr}{\int \rho_-(r)dr} = (x_-, y_-, z_-) \quad \text{....................... (S6)}$$

The spatial distance (D_{CT}) between the two barycenters R_+ and R_- of density distributions can thus be used to measure the CT excitation length

$$D_{CT} = |R_+ - R_-| \text{.................. (S7)}$$

The transferred charge (q_{CT}) can be obtained by integrating over all space $\rho_+ (\rho_-)$,. Variation in dipole moment between the ground and the excited states (μ_{CT}) can be computed by the following relation:

$$\|\mu_{CT}\| = D_{CT}\int \rho_+(r)dr = D_{CT}\int \rho_-(r)dr \text{......................... (S8)}$$

$$= D_{CT}q_{CT} \text{................. (S9)}$$

The difference between the dipole moments $\|\mu_{CT}\|$ have been computed for the ground and the excited states $\Delta\mu_{ES-GS}$. The two centroids of charges (C^+/C^-) associated to the positive and negative density regions are calculated as follows. First the root–mean–square deviations along the three axis (σ_{aj}, j = x, y, z; a = + or –) are computed as

$$\sigma_{a,j} = \sqrt{\frac{\sum_i \rho_a(r_i)(j_i - j_a)^2}{\sum_i \rho_a(r_i)}} \text{........................... (S10)}$$

The two centroids (C_+ and C_-) are defined as

$$C_+(r) = A_+ e\left(-\frac{(x - x_+)^2}{2\sigma_{+x}^2} - \frac{(y - y_+)^2}{2\sigma_{+y}^2} - \frac{(z - z_+)^2}{2\sigma_{+z}^2}\right) \text{.................... (S11)}$$

$$C_{-+}(r) = A_- e\left(-\frac{(x - x_-)^2}{2\sigma_{-x}^2} - \frac{(y - y_-)^2}{2\sigma_{-y}^2} - \frac{(z - z_-)^2}{2\sigma_{-z}^2}\right) \text{.................... (S12)}$$

The normalization factors (A+ and A_) are used to impose the integrated charge on the centroid to be equal to the corresponding density change integrated in the whole space:

$$A_{+} = \frac{\int \rho_{+}(r)dr}{\int e(-\frac{(x - x_{-})^{2}}{2\sigma_{+x}^{2}} - \frac{(y - y_{-})^{2}}{2\sigma_{+y}^{2}} - \frac{(z - z_{-})^{2}}{2\sigma_{+z}^{2}})dr} \quad \text{.................. (S13)}$$

$$A_{-} = \frac{\int \rho_{-}(r)dr}{\int e(-\frac{(x - x_{-})^{2}}{2\sigma_{-x}^{2}} - \frac{(y - y_{-})^{2}}{2\sigma_{-y}^{2}} - \frac{(z - z_{-})^{2}}{2\sigma_{-z}^{2}})dr} \quad \text{.................. (S14)}$$

H index is defined as half of the sum of the centroids axis along the D–A direction, if the D–A direction is along the X axis, H is defined by the relation:

$$H = \frac{\sigma_{+x} + \sigma_{-x}}{2} \quad \text{.................... (S15)}$$

The centroid along X axis is expected. The t intex represents the difference between D_{CT} and H:

$$t = D_{CT} - H \quad \text{............... (S16)}$$

Figure S1. Potential energy surface scan (PES) diagram of (a) Cz-DEPVI and (b)TPA-DEPVI and (c) Molecular electrostatic potential (ESP) surface of Cz-DPVI, Cz- DMPVI, Cz-DEPVI and TPA-DEPVI.

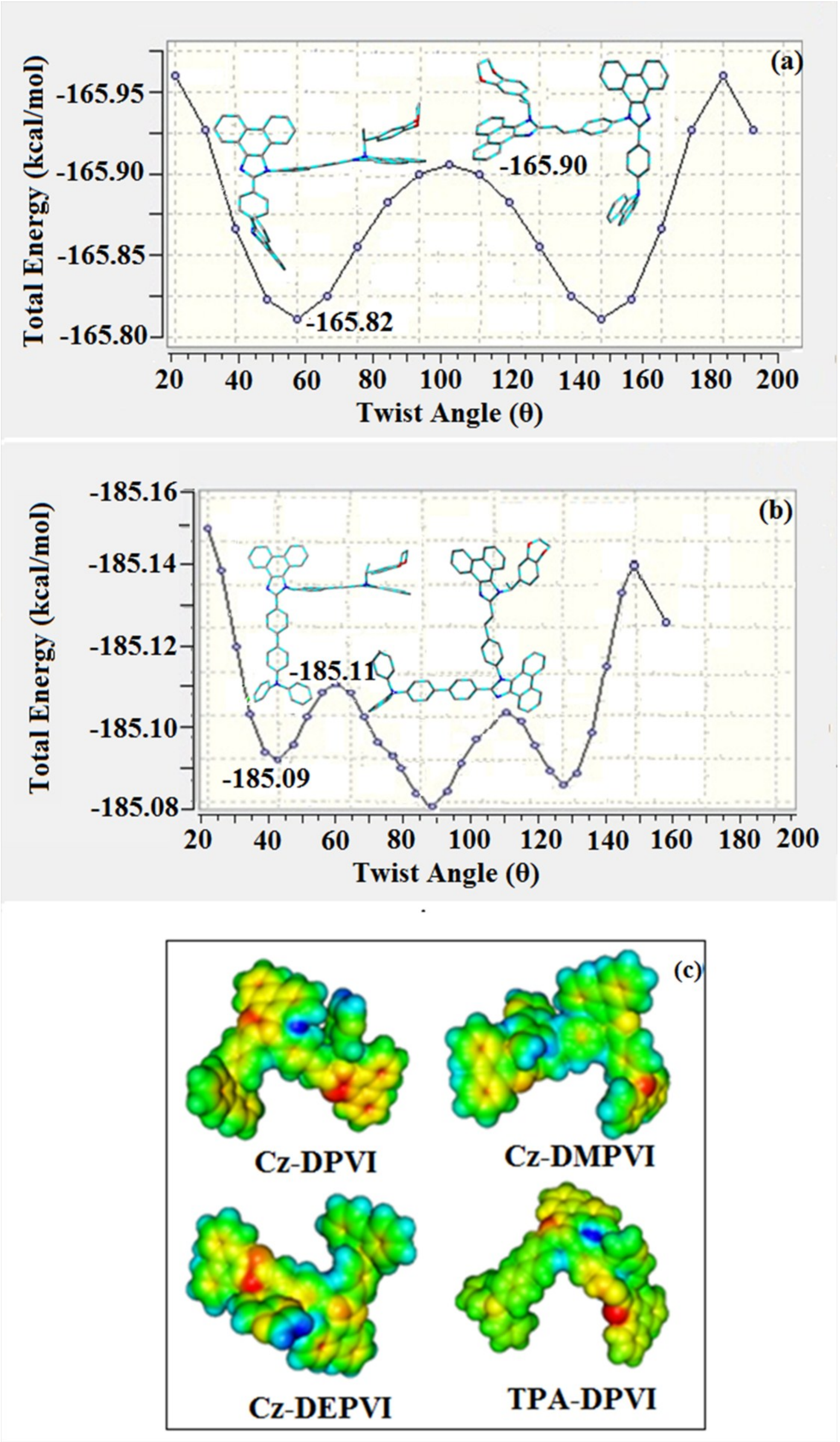

Figure S2. Molecular structure, Optimized geometry and HOMO and LUMO of Cz-DPVI, Cz-DMPVI, Cz-DEPVI and TPA-DEPVI

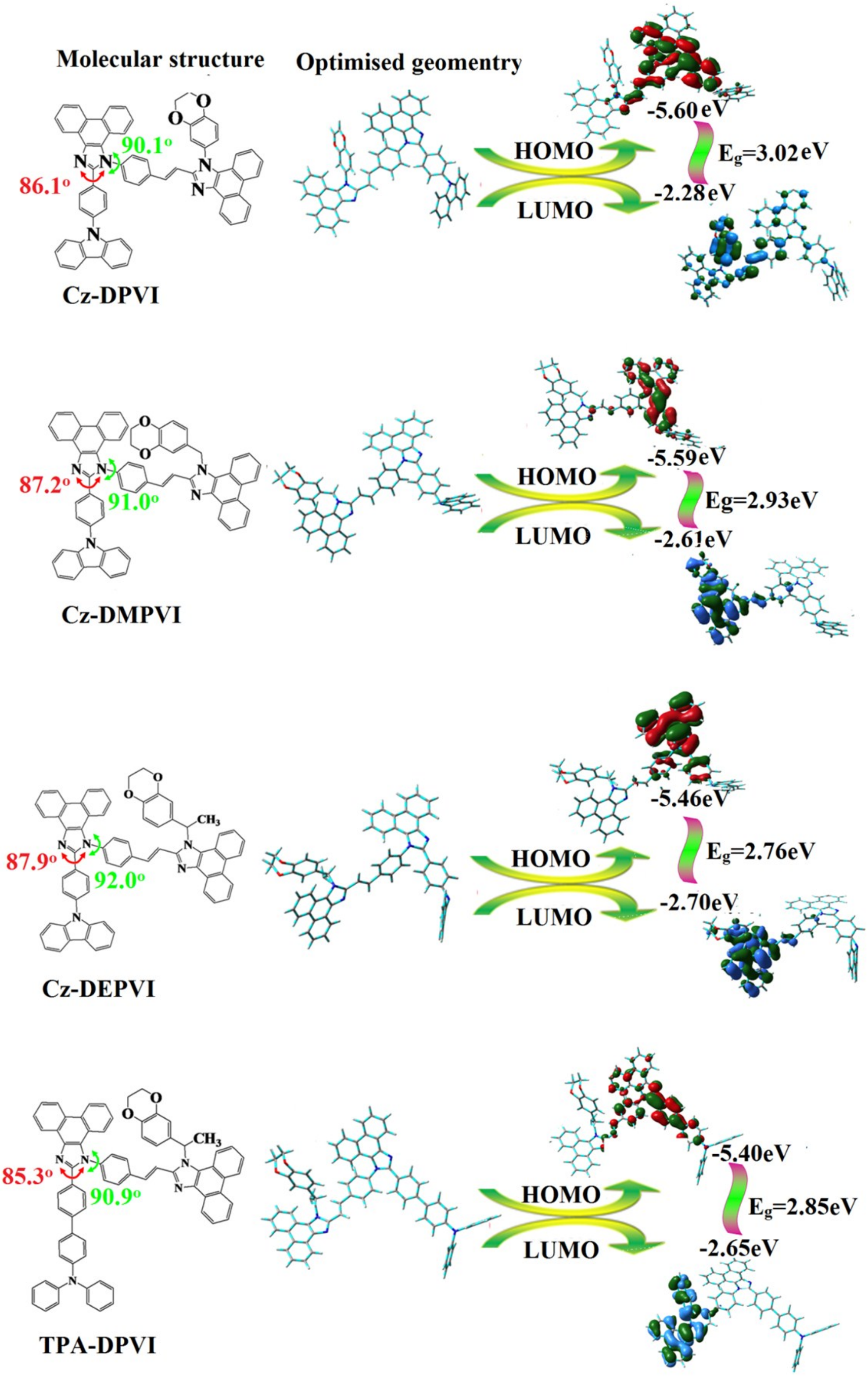

Figure S3. Solvatochromic emission spectra of a) Cz-DEPVI and b) TPA-DEPVI (inset: fluorescence and phosphorescence spectra of Cz-DEPVI and TPA-DEPVI in CH_2Cl_2 at low temperature)

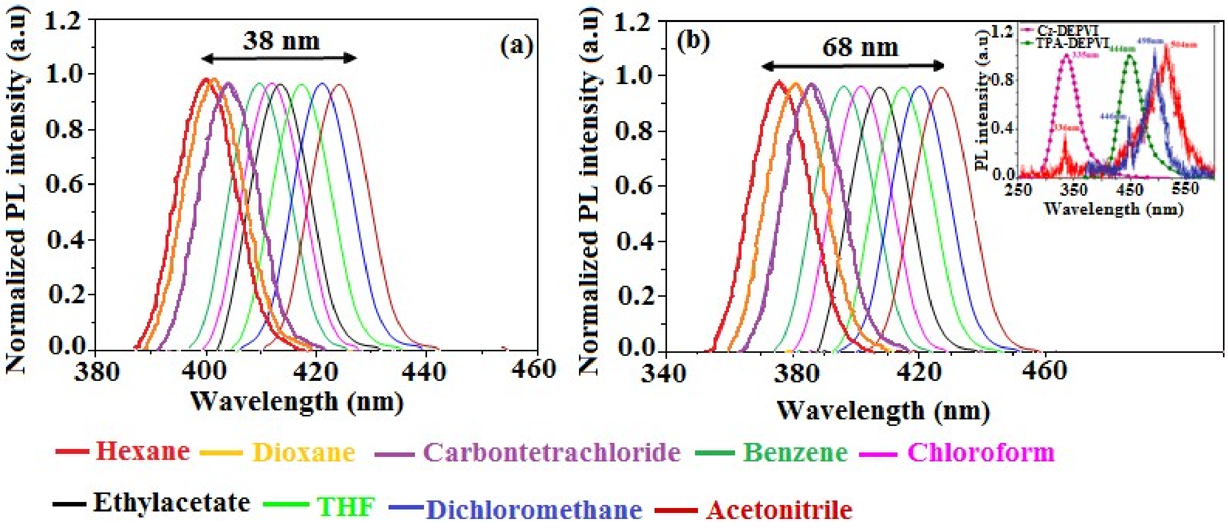

Figure S4. Normalized absorption spectra of a) Cz-DEPVI and b) TPA-DEPVI

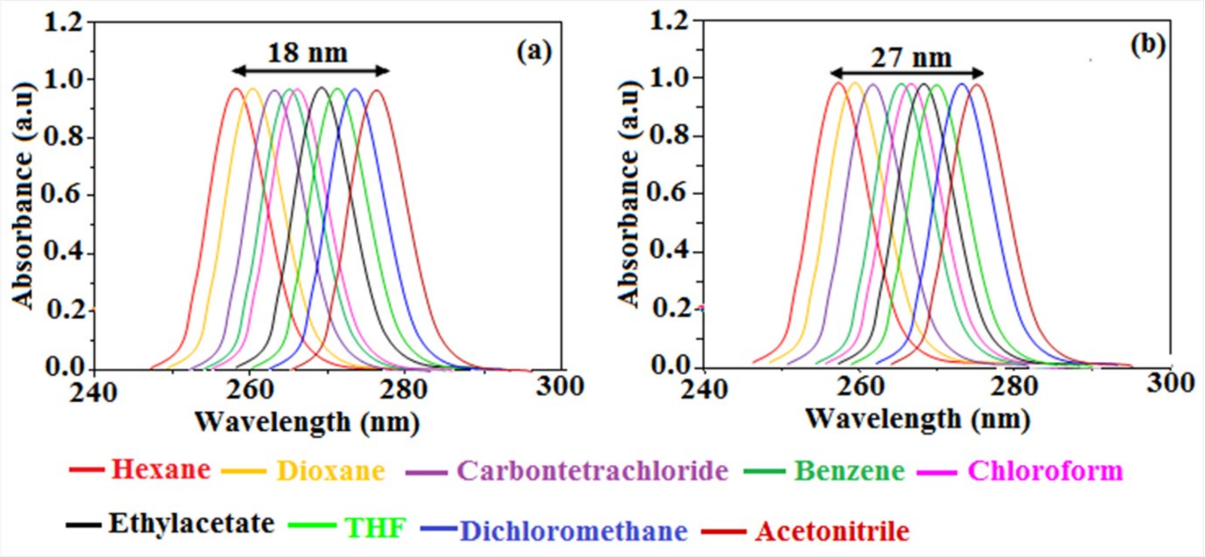

Figure S5. Natural transition orbital (NTO) pairs with transition character analysis for singlet states (S_1-S_{10}) and triplet states (T_1-T_{10}) of TPA-DEPVI [*f*-oscillator strength and % weights of hole-particle].

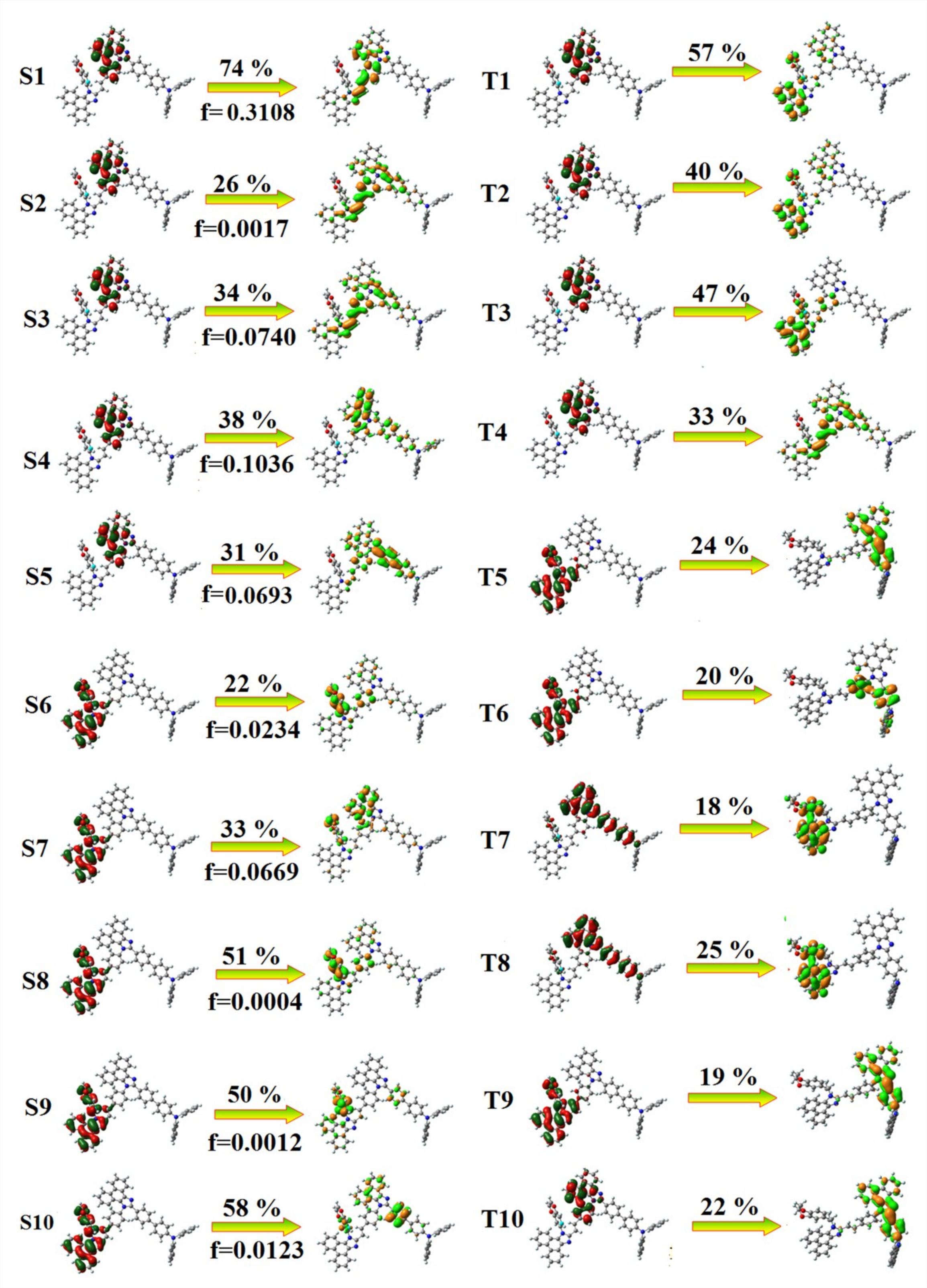

Figure S6. Hole and particle distribution of TPA-DEPVI [S_1–S_5 states: ● -green increasing electron density and ● - blue decreasing electron density (density=transition=n IOp (6/8=3)].

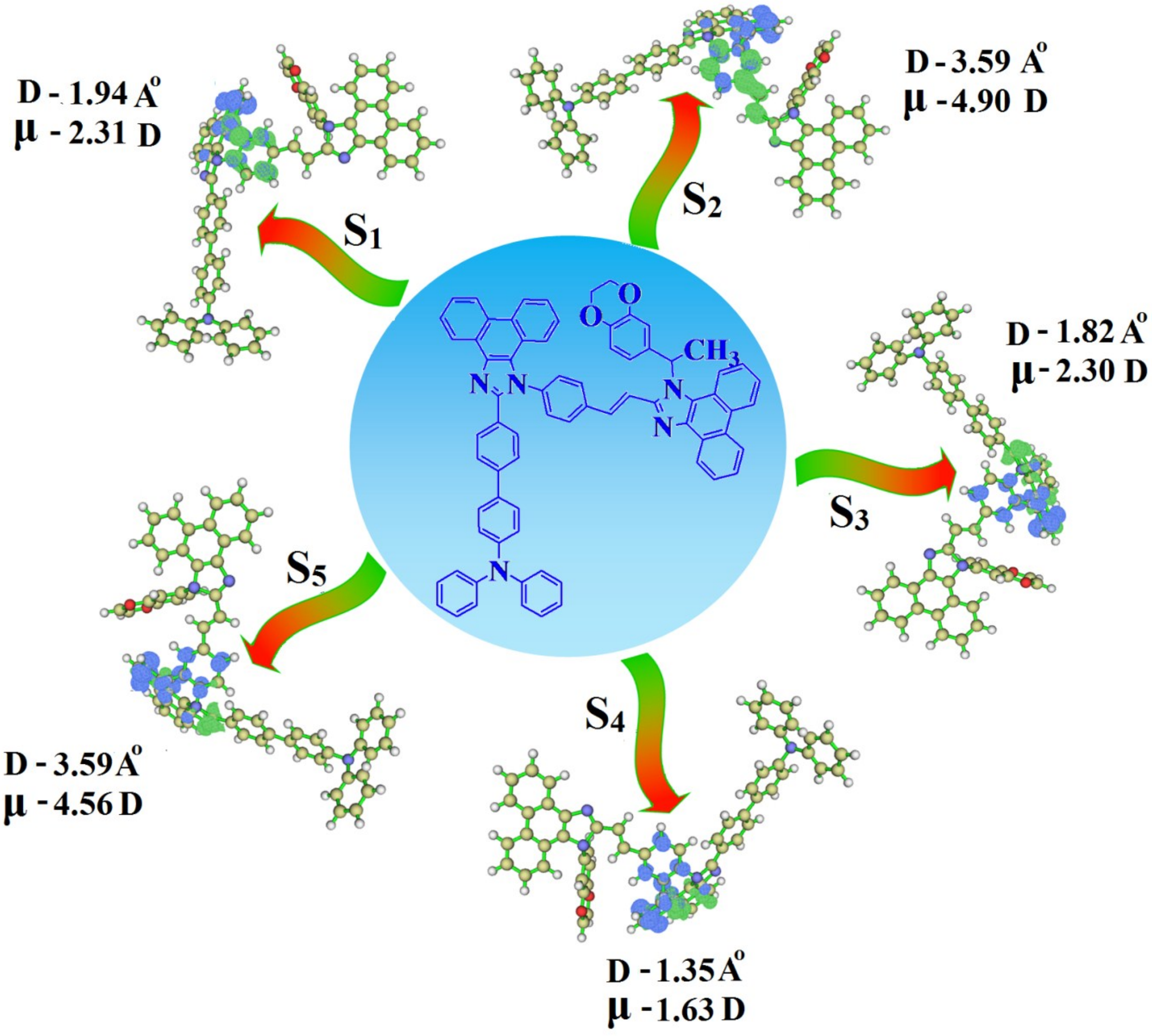

Figure S7: Computed contour plots of transition density matrices (TDM) of TPA-DEPVI for [S_1–S_5 states: density=transition=n /IOp(6/8=3)].

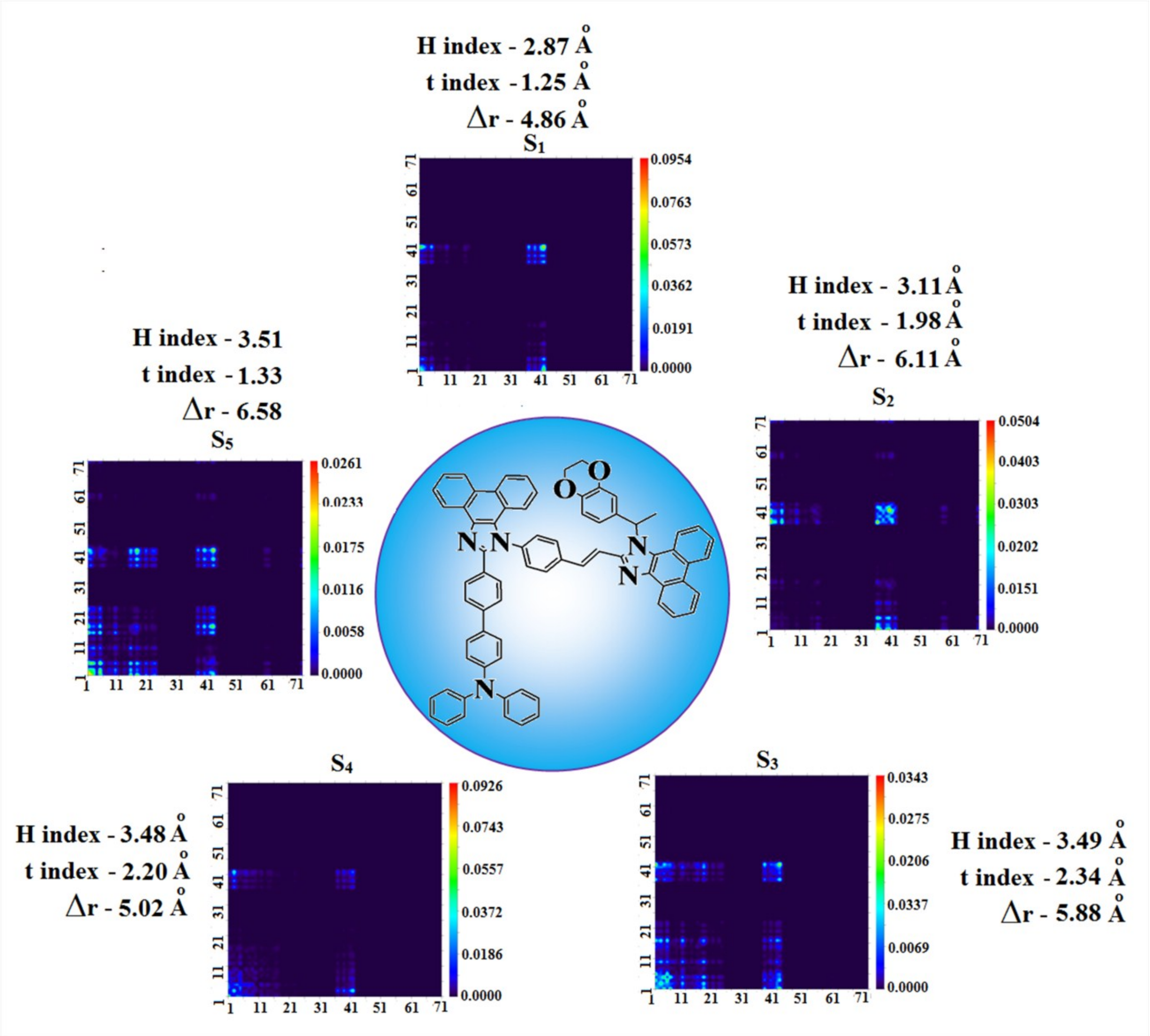

Table S1: Photophysical properties of Cz-DEPVI in different solvents.

Solvents	ε	n	f(ε,n)	ET(30)	λ_{ab} (nm)	ν_{ab} (cm^{-1})	λ_{flu} (nm)	ν_{flu} (cm^{-1})	ν_{ss} (cm^{-1})	ΔG (kcal/mol)	$\Delta(\Delta G_{hex}-\Delta G_{sol})$ (kcal/mol)	λ (kcal/mol)
Hexane	1.88	1.37	0.000411	32.4	260	38461.54	399	25062.66	13398.88	90.79	0.00	19.15
Dioxane	2.2	1.42	0.021437	36	268	37313.43	409	24449.88	12863.56	88.28	2.51	18.39
Carbontetrachloride	2.23	1.46	0.011075	39.1	273	36630.04	411	24330.9	12299.14	87.13	3.66	17.58
Benzene	2.28	1.42	0.026639	34.3	262	38167.94	413	24213.08	13954.86	89.16	1.63	19.95
Chloroform	4.81	1.44	0.148262	39.1	271	36900.37	417	23980.82	12919.55	87.01	3.78	18.47
Ethyl acetate	6.09	1.41	0.186569	38.1	267	37453.18	421	23752.97	13700.21	87.48	3.31	19.58
THF	7.52	1.40	0.209634	37.4	265	37735.85	424	23584.91	14150.94	87.64	3.15	20.23
Dichloromethane	9.08	1.42	0.218349	40.7	260	38461.54	428	23364.49	15097.05	88.37	2.42	21.58
Acetonitrile	37.5	1.34	0.305378	45.6	278	35971.22	437	22883.3	13087.93	84.12	6.67	18.71

Table S2: Photophysical properties of TPA-DEPVI in different solvents.

**Solvents**	**ε**	**n**	**f(ε,n)**	**ET(30)**	**λ_{ab} (nm)**	**ν_{ab} (cm^{-1})**	**λ_{flu} (nm)**	**ν_{flu} (cm^{-1})**	**ν_{ss} (cm^{-1})**	**ΔG (kcal/mol)**	**$\Delta(\Delta G_{hex}-\Delta G_{sol})$ (kcal/mol)**	**λ (kcal/mol)**
Hexane	1.88	1.37	0.000411	32.4	289	34602.08	380	26315.79	8286.287	87.07	3.72	11.84
Dioxane	2.22	1.42	0.021437	36	268	37313.43	409	24449.88	12863.56	88.28	2.51	18.39
Carbontetrachloride	2.23	1.46	0.011075	39.1	273	36630.04	411	24330.9	12299.14	87.13	3.66	17.58
Benzene	2.28	1.42	0.026639	34.3	262	38167.94	413	24213.08	13954.86	89.16	1.63	19.95
Chloroform	4.81	1.44	0.148262	39.1	271	36900.37	417	23980.82	12919.55	87.01	3.78	18.47
Ethyl acetate	6.09	1.41	0.186569	38.1	267	37453.18	421	23752.97	13700.21	87.48	3.31	19.58
THF	7.52	1.40	0.209634	37.4	265	37735.85	424	23584.91	14150.94	87.64	3.15	20.23
Dichloromethane	9.08	1.42	0.218349	40.7	255	39215.69	432	23148.15	16067.54	89.13	1.66	22.96
Acetonitrile	37.5	1.34	0.305378	45.6	262	38167.94	448	22321.43	15846.51	86.45	4.34	22.65

Table S3. Percentage transition of LE and CT of Cz-DEPVI and TPA-DEPVI

**% of transition**	**Cz-DEPVI**		**TPA-DEPVI**	
	**Singlet (S_1-S_{10})**	**Triplet (T_1-T_{10})**	**Singlet (S_1-S_{10})**	**Triplet (T_1-T_{10})**
% CT	70	65	80	70
% LE	30	35	20	30

Table S4. Computed [zindo (Singlet or Triplet, n states=10)] singlet (E_S) and triplet (E_T) energies, oscillator strength (*f*), dipole moment (μ) and singlet-triplet energy difference (ΔE_{S-T}) of Cz-DEPVI from NTOs.

Energy level	E_S (eV)	Oscillator strength (f)	μ (D)	NTO Transitions	E_T (eV)	ΔE_{S-T}(eV)	NTO Transitions
1	1.68	0.6774	2.3747	$171 \xrightarrow{78\%} 182$	0.47	1.21	$174 \xrightarrow{34\%} 181$
2	2.57	0.0908	0.6377	$173 \xrightarrow{41\%} 174$	1.67	0.90	$170 \xrightarrow{23\%} 174$
3	3.02	0.4391	0.8996	$173 \xrightarrow{33\%} 174$	1.69	1.23	$170 \xrightarrow{21\%} 175$
4	3.34	0.2012	2.3864	$173 \xrightarrow{51\%} 176$	1.98	1.36	$171 \xrightarrow{19\%} 176$
5	3.37	0.5731	1.0767	$170 \xrightarrow{68\%} 175$	2.08	1.29	$171 \xrightarrow{25\%} 175$
6	3.41	0.3183	1.7652	$172 \xrightarrow{24\%} 174$	2.23	1.18	$174 \xrightarrow{43\%} 177$
7	3.52	0.0026	0.8435	$169 \xrightarrow{22\%} 174$	2.58	0.94	$174 \xrightarrow{21\%} 177$
8	3.63	0.0728	0.5623	$170 \xrightarrow{36\%} 177$	2.80	0.83	$174 \xrightarrow{56\%} 175$
9	3.67	0.0855	1.2258	$172 \xrightarrow{33\%} 176$	2.97	0.70	$174 \xrightarrow{37\%} 175$
10	3.74	0.0309	1.8824	$172 \xrightarrow{25\%} 182$	3.04	0.70	$174 \xrightarrow{26\%} 181$

Table S5: Computed excitation energy (eV), excitation coefficient and Δr intex (Å) for ten singlet & triplet states of Cz-DEPVI

State	Singlet			Triplet		
	Excitation energy	Excitation coefficient	Δr intex	Excitation energy	Excitation coefficient	Δr intex
1	1.6792	0.4560	6.1302	0.4692	0.4489	2.1246
2	2.5693	0.3486	9.1283	1.6392	0.4253	2.6801
3	3.0248	0.3508	8.3764	1.6880	0.3575	2.0912
4	3.3440	0.3759	2.0683	1.9820	0.3830	1.7632
5	3.3665	0.4013	5.8056	2.0829	0.4415	4.9411
6	3.4096	0.3801	9.7733	2.2381	0.4256	5.9639
7	3.5196	0.3590	5.7968	2.5758	0.4209	3.0534
8	3.6268	0.3976	8.5321	2.8002	0.4199	4.5849
9	3.6651	0.3534	4.5477	2.9689	0.2959	3.7891
10	3.7438	0.2745	9.0096	3.0353	0.3444	1.9535

Table S6: Computed excitation energy (eV), excitation coefficient and Δr intex (Å) for ten singlet & triplet states of TPA-DEPVI

State	Singlet			Triplet		
	**Excitation energy**	**Excitation coefficient**	**Δr intex**	**Excitation energy**	**Excitation coefficient**	**Δr intex**
1	0.5681	0.4478	4.8648	0.2481	0.4466	1.8351
2	1.1973	0.4645	6.1118	0.5681	0.3178	2.3401
3	1.3078	0.4466	5.8811	1.6056	0.2637	2.5616
4	1.9308	0.4446	5.0247	1.8630	0.3443	3.8946
5	2.1224	0.4569	6.5831	2.0123	0.4784	2.5931
6	2.4280	0.4443	6.6374	2.1034	0.1120	3.3011
7	2.5843	0.4122	5.6830	2.1270	0.2803	2.6892
8	2.758	0.4664	7.8372	2.1847	0.2717	3.3942
9	2.9059	0.4683	8.0853	2.4971	0.2637	2.4448
10	2.9878	0.4371	6.6772	2.6018	0.1361	3.4152

Table S7: Computed RMSD of electron and hole, H index and t index for ten singlet states (S_1–S_{10}) of Cz-DEPVI

State	Electron RMSD				Hole RMSD				H index				t index			
	x	y	z	total	x	y	z	total	x	y	z	Total	x	y	z	Total
S1	2.278	1.371	0.870	2.797	1.120	0.905	0.704	1.603	1.699	1.138	0.787	2.191	-1.590	-0.970	-0.495	1.927
S2	0.640	1.647	1.077	3.877	1.622	1.755	1.072	2.619	2.481	1.701	1.075	3.194	0.771	-1.564	-0.290	1.768
S3	3.194	2.017	1.134	3.944	2.973	1.975	1.348	3.815	3.084	1.996	1.241	3.877	-0.862	-1.858	-1.191	2.369
S4	1.968	2.421	1.805	3.604	2.038	1.980	1.460	3.194	2.003	2.200	1.632	3.394	-1.618	-1.883	-1.618	2.963
S5	2.375	2.964	0.936	3.911	2.175	2.466	0.940	3.420	2.275	2.715	0.938	3.664	-1.594	-0.720	-0.613	1.853
S6	3.497	2.249	1.293	4.354	1.818	1.913	1.487	3.029	2.657	2.081	1.390	3.650	-0.072	-1.410	-1.081	1.778
S7	3.529	2.002	1.143	4.215	4.225	2.140	1.216	4.890	3.877	2.071	1.179	4.551	-2.554	-0.636	-0.956	2.800
S8	2.479	2.192	0.978	3.451	2.211	2.520	0.977	3.492	2.345	2.356	0.977	3.465	-1.849	-1.660	-0.848	2.625
S9	2.653	2.543	1.604	4.009	2.501	1.885	1.452	3.452	2.577	2.214	1.528	3.725	-2.270	-1.635	-0.728	2.891
S10	4.002	2.217	1.190	4.727	4.769	2.340	1.353	5.482	4.386	2.278	1.271	5.103	-3.044	-1.865	-1.151	3.751

Table S8: Computed RMSD of electron and hole, H index and t index for first ten singlet states (S_1–S_{10}) of TPA-DEPVI

State	Electron RMSD				Hole RMSD				H index				t index			
	**x**	**y**	**z**	**total**	**x**	**y**	**z**	**total**	**x**	**y**	**z**	**Total**	**x**	**y**	**z**	**Total**
S1	1.736	2.145	0.864	2.892	1.669	2.136	0.907	2.858	1.703	2.140	0.886	2.875	-0.973	-0.346	-0.719	1.259
S2	2.164	2.466	0.803	3.378	1.669	2.136	0.907	2.858	1.917	2.301	0.855	3.114	-1.403	1.252	-0.629	1.983
S3	2.899	2.813	0.965	4.153	1.669	2.136	0.907	2.858	2.284	2.474	0.936	3.495	-0.535	-2.236	-0.484	2.349
S4	2.753	2.835	1.149	4.115	1.669	2.136	0.907	2.858	2.211	2.485	1.028	3.482	-1.195	-1.799	-0.459	2.208
S5	3.326	2.520	0.795	4.248	1.669	2.136	0.907	2.858	2.497	2.328	0.851	3.519	0.798	-0.948	-0.491	1.333
S6	3.848	2.792	0.814	4.824	1.669	2.136	0.907	2.858	2.759	2.464	0.860	3.797	-1.853	2.622	-0.317	3.227
S7	1.877	2.689	1.211	3.496	1.669	2.136	0.907	2.858	1.773	2.412	1.059	3.176	-0.403	-0.326	0.165	0.544
S8	2.677	1.947	0.937	3.440	1.669	2.136	0.907	2.858	2.173	2.041	0.922	3.121	1.591	1.879	-0.339	2.486
S9	1.716	1.547	1.170	2.589	1.669	2.136	0.907	2.858	1.692	1.841	1.038	2.708	2.318	2.330	-0.465	3.319
S10	3.441	1.634	0.850	3.902	1.669	2.136	0.907	2.858	2.555	1.885	0.879	3.294	2.520	0.652	-0.494	2.649

Table S9: Computed hole and electron overlap (S), distance between centroids of hole and electron (D, Å) and dipole moment (μ) for ten S_1–S_{10} states of Cz-DEPVI

State	Hole integral	Electron integral	Integral of transition density	Integral overlap of hole & electron (S)	Centroid of hole (Å)			Centroid of electron (Å)			D (Å)	μ (a.u)
					x	y	z	x	y	z		
S1	0.7837	0.5790	0.0058	0.1593	-9.9045	2.5856	-0.1954	-10.0133	2.4174	0.0966	0.35	0.46
S2	0.6402	0.4953	-0.0100	0.1226	-5.2153	-0.1879	-1.3199	-1.9634	-0.3246	-0.5357	3.34	3.59
S3	0.6802	0.4964	0.0228	0.2185	-4.7702	-0.6418	-0.7301	-2.5485	-0.5040	-0.6796	2.22	2.48
S4	0.7722	0.5743	-0.0002	0.3394	-6.1472	-1.4003	-0.1825	-6.5321	-1.7180	-0.1686	0.49	0.64
S5	0.8288	0.6351	-0.0088	0.3123	4.1802	4.3314	-0.1969	4.8610	2.3361	0.1280	2.13	2.95
S6	0.7594	0.5683	0.0159	0.2192	-6.2481	-0.3690	-0.2180	-3.6625	-1.0400	-0.5270	2.68	3.38
S7	0.7206	0.5750	-0.0053	0.2592	-0.8634	0.5948	-0.3187	-2.1870	-0.8403	-0.5419	1.96	2.40
S8	0.8356	0.6104	-0.0012	0.4155	4.1910	4.6030	-0.2876	3.6943	5.2991	-0.4169	0.86	1.18
S9	0.6874	0.5337	0.0015	0.2814	-5.9464	0.3523	-0.4314	-6.2531	-0.2271	0.3686	1.03	1.19
S10	0.5663	0.4238	-0.0079	0.2396	-1.8143	0.3013	-0.1166	-0.4726	-0.1115	-0.2365	1.40	1.32

Table S10: Computed hole and electron overlap (S), distance between centroids of hole and electron (D, Å) and dipole moment (μ) for ten S_1–S_{10} states of TPA-DEPVI

State	Hole integral	Electron integral	Integral of transition density	Integral overlap of hole & electron (S)	Centroid of hole (Å)			Centroid of electron (Å)			D (Å)	μ (a.u)
					x	y	z	x	y	z		
S1	0.6946	0.5665	-0.0364	0.2669	-2.12	4.10	-0.64	-1.39	2.30	-0.80	1.94	2.31
S2	0.7206	0.7228	-0.0027	0.1458	-2.12	4.10	-0.64	-1.61	0.54	-0.41	3.59	4.90
S3	0.6928	0.6450	-0.0449	0.1712	-2.12	4.10	-0.64	-0.37	3.86	-0.18	1.82	2.30
S4	0.6897	0.5888	0.0651	0.2505	-2.12	4.10	-0.64	-1.11	4.78	-0.07	1.35	1.63
S5	0.7089	0.6368	-0.0342	0.1756	-2.12	4.10	-0.64	1.16	2.72	-0.28	3.59	4.56
S6	0.6892	0.6594	-0.0295	0.0826	-2.12	4.10	-0.64	-3.03	-0.98	-0.09	5.19	6.61
S7	0.6395	0.5555	0.0037	0.1330	-2.12	4.10	-0.64	-0.75	6.18	0.58	2.77	3.13
S8	0.7235	0.6588	0.0051	0.0112	-2.12	4.10	-0.64	-5.89	0.18	-0.05	5.46	7.13
S9	0.7264	0.6611	-0.0063	0.0108	-2.12	4.10	-0.64	-6.13	-0.06	-0.06	5.81	7.62
S10	0.6781	0.5431	-0.0100	0.0334	-2.12	4.10	-0.64	2.94	1.56	-0.25	5.68	6.56

Table S11. Transferred charges (q_{CT}), barycentres of electron density loss (R_+) /gain (R-), distance between two barycenters (D_{CT}), dipole moment of CT (μ_{CT}), RMSD of +ve/-ve parts, CT indices (H & t) and overlap integral of C+/C- of Cz-DPVI, Cz-DMPVI, Cz-DEPVI and TPA-DEPVI

**Blue emissive &Host materials**	$\mathbf{q_{CT}}$ $\lvert e^{-1}\rvert$	$\mathbf{R_+}$ **(Å)**			**R- (Å)**			$\mathbf{D_{CT}}$ **(Å)**	$\boldsymbol{\mu_{CT}}$ **(D)**	**RMSD of +ve parts**	**RMSD of -ve parts**	**H / t indices (Å)**	**overlap integral of C+/ C-**
		x	y	z	x	y	z						
Cz-DPVI	301.100-312.814	-0.73	-0.19	-0.01	0.56	0.37	0.01	1.422	402.18	12.66	13.34	6.87/5.52	0.9802
Cz-DMPVI	319.523-332.543	0.87	-0.00	0.16	1.03	-0.47	-0.33	0.703	436.75	13.07	13.47	7.02/6.55	0.9814
Cz-DEPVI	338.012-342.598	-0.08	-0.40	0.58	0.44	-0.07	-0.19	1.008	542.12	14.49	14.35	7.62/6.86	0.9694
TPA-DEPVI	559.680-562.366	-0.93	-1.27	0.16	-0.29	-1.08	0.25	0.352	608.69	15.90	16.12	8.46/8.12	0.9718
